# FI-Net: Identification of Cancer Driver Genes by Using Functional Impact Prediction Neural Network

**DOI:** 10.3389/fgene.2020.564839

**Published:** 2020-11-10

**Authors:** Hong Gu, Xiaolu Xu, Pan Qin, Jia Wang

**Affiliations:** ^1^Faculty of Electronic Information and Electrical Engineering, Dalian University of Technology, Dalian, China; ^2^Department of Breast Surgery, Institute of Breast Disease, Second Hospital of Dalian Medical University, Dalian, China

**Keywords:** cancer research, driver genes, functional impact, artificial neural network, multi-omics features, hierarchical clustering algorithm

## Abstract

Identification of driver genes, whose mutations cause the development of tumors, is crucial for the improvement of cancer research and precision medicine. To overcome the problem that the traditional frequency-based methods cannot detect lowly recurrently mutated driver genes, researchers have focused on the functional impact of gene mutations and proposed the function-based methods. However, most of the function-based methods estimate the distribution of the null model through the non-parametric method, which is sensitive to sample size. Besides, such methods could probably lead to underselection or overselection results. In this study, we proposed a method to identify driver genes by using functional impact prediction neural network (FI-net). An artificial neural network as a parametric model was constructed to estimate the functional impact scores for genes, in which multi-omics features were used as the multivariate inputs. Then the estimation of the background distribution and the identification of driver genes were conducted in each cluster obtained by the hierarchical clustering algorithm. We applied FI-net and other 22 state-of-the-art methods to 31 datasets from The Cancer Genome Atlas project. According to the comprehensive evaluation criterion, FI-net was powerful among various datasets and outperformed the other methods in terms of the overlap fraction with Cancer Gene Census and Network of Cancer Genes database, and the consensus in predictions among methods. Furthermore, the results illustrated that FI-net can identify known and potential novel driver genes.

## 1. Introduction

Cancers have been known to be caused by the accumulation of mutations throughout the life of an individual. Next-generation sequencing (Goodwin et al., 2016) technology provides a new perspective on cancer research. Genomics sequencing data across all major cancer types are available from a variety of cancer sequencing projects, such as International Cancer Genome Consortium (Hudson et al., 2010) and The Cancer Genome Altas (TCGA) (Weinstein et al., 2013). A tremendous challenge is to distinguish driver genes with mutations that are involved in tumorigenesis. Sufficient identification of driver genes promotes the understanding of tumor progression and ensures the efficiency of gene-targeted therapy for cancers (Chin et al., 2011; Shin et al., 2017).

Nowadays, numerous methods for identifying cancer driver genes have been proposed. The frequency-based methods, which pick out driver genes by counting the mutations in a cohort of patients, were first developed. MuSiC (Dees et al., 2012) and MutSigCV (Lawrence et al., 2013) are two popular frequency-based methods. The main differences between these two methods are the statistics of the hypothesis test and the procedures for estimating the background mutation rate. Some other frequency-based methods have also been studied rapidly after them, such as Lanzos et al. (2017) and Han et al. (2019). Note that such methods did not take genetic functions of mutations into consideration. Thus, they have some known limitations, such as a high false positive rate, and they often fail to detect driver genes with low mutation frequencies (Bashashati et al., 2012; Koboldt et al., 2012; Muzny et al., 2012). Based on the hypothesis that gene mutations tend to converge on a few biological pathways, some pathway-based methods attempt to identify cancer driver modules consisting of multiple genes rather than individual genes using some biological prior knowledge (Bashashati et al., 2012; Paull et al., 2013; Leiserson et al., 2015; Gao et al., 2017; Hou et al., 2018; Carlin et al., 2019). However, the application of these methods is limited by the incompleteness of prior knowledge database.

The functional impacts of gene mutations reflect how the mutations affect protein function and hence, potentially alter the phenotype (Ng and Henikoff, 2003). To improve the sensitivity to driver genes with low mutation frequencies, the function-based methods that identify genes by assessing their bias toward the accumulation of mutations with high functional impact were proposed (Gonzalezperez and Lopezbigas, 2012; Ryslik et al., 2013; Tamborero et al., 2013a; Jia et al., 2014; Portapardo and Godzik, 2014; Mularoni et al., 2016; Wang et al., 2018). For example, MSEA predicted driver genes by assessing whether a protein domain has a higher mutation rate than the remaining region of the protein (Jia et al., 2014). OncodriveFML identified driver genes by comparing the average functional impact score observed in each genomic region to the expected score calculated by random sampling (Mularoni et al., 2016). rDriver developed a Bayesian framework to detect driver genes based on both the functional impact of mutations and the genome-wide expression levels (Wang et al., 2018). The advantage of these methods is that the identified driver genes show positive selection on protein level rather than just mutation level. However, the experimental results showed that function-based methods can still be improved. Some function-based methods exhibit overselection, that is, detecting too many driver genes. For example, MSEA (Jia et al., 2014) identified 2,003 driver genes in pancreatic adenocarcinoma, and iPAC (Ryslik et al., 2013) identified 16,799 driver genes in liver hepatocellular carcinoma. Furthermore, the distributions of the null model in most of the function-based methods were estimated using the non-parametric methods (e.g., Gonzalezperez and Lopezbigas, 2012; Tamborero et al., 2013a; Jia et al., 2014; Portapardo and Godzik, 2014; Mularoni et al., 2016), which could make the methods sensitive to sample size (Whitley and Ball, 2002).

To tackle the problems mentioned above, we propose a novel function-based method FI-net to identify driver genes. The somatic mutation frequency of genes is affected by several factors and varies across the genomic sequence (Martincorena et al., 2012; Roberts et al., 2012). By making a similar hypothesis, we first constructed an artificial neural network (ANN) model to estimate the functional impact scores (FISs) of genes by using genetic features from multi-omics data sources. The R-squared for the ANN regression model in the 31 TCGA datasets ranged from 0.5391 (brain lower grade glioma) to 0.9673 (colorectal adenocarcinoma) with the mean being 0.8748. To evaluate the local distribution of background functional impact score (BFIS), we then clustered genes in the multi-omics feature space using the hierarchical clustering algorithm. A gamma distribution was further fitted in each cluster to obtain the background distribution. Finally, the observed FISs were compared to the background distribution to obtain the empirical *p*-values for genes within each cluster. For multiple testing, *q*-values were assigned to genes using the false discovery rate approach. Genes that show significant bias (*q*-value ≤ 0.05) were selected as driver genes in a cohort of patients. To the best of our knowledge, this study is the first research to build a mathematical model for estimating the background distribution of gene functional impact. We applied FI-net to the 31 TCGA datasets to verify the performance of identifying driver genes. Overall, FI-net detected the adequate number of driver genes in the 31 datasets. The identified driver genes showed high deleterious mutation ratio and high coverage in a cohort of patients and were enriched for known cancer driver genes included in the Cancer Gene Census (CGC) database (Futreal et al., 2004) and the Network of Cancer Genes (NCG) database (Repana et al., 2019). Moreover, we demonstrated that FI-net can identify potential novel driver genes.

## 2. Materials and Methods

**Figure 1:**
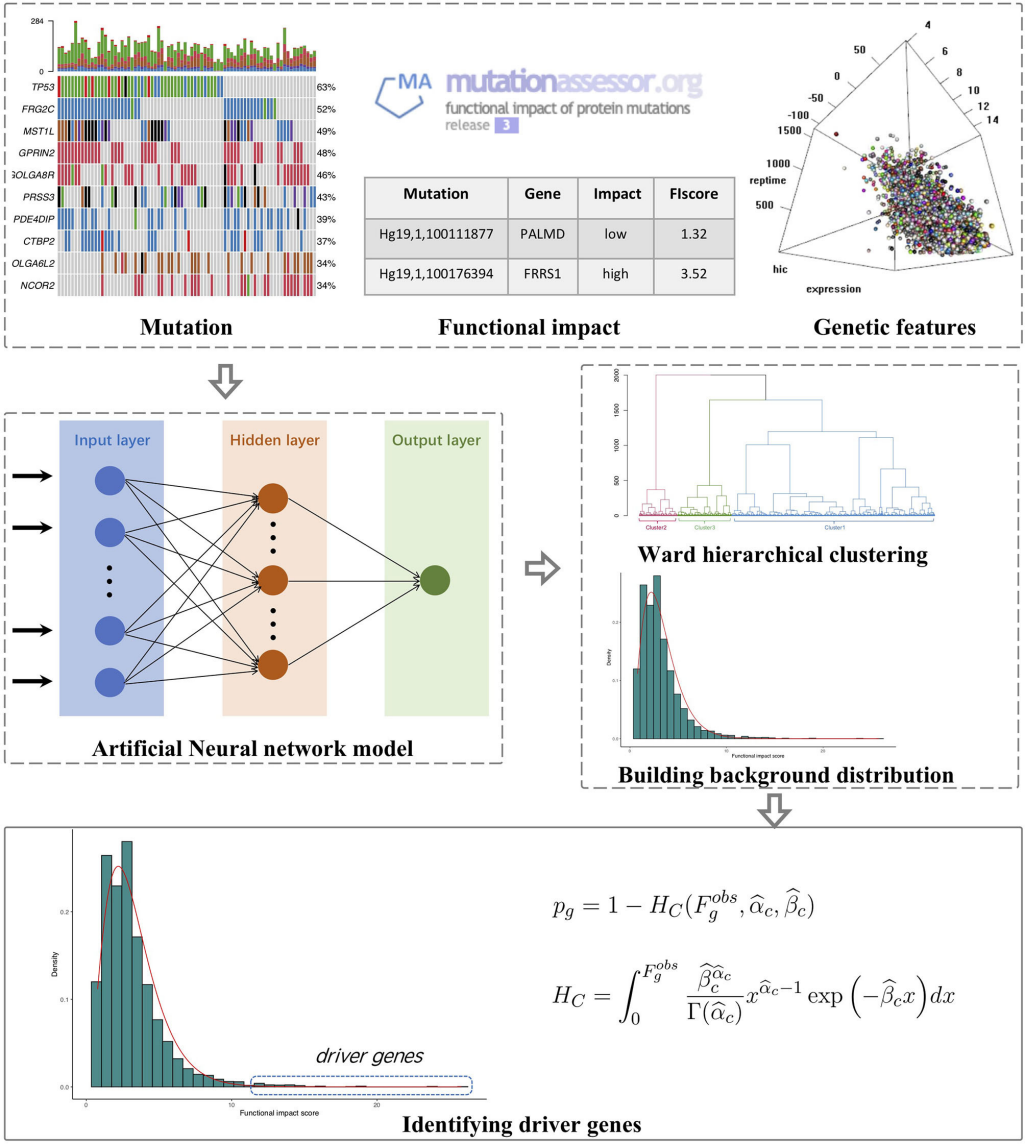
The workflow of functional impact prediction neural network (FI-net) method. The observed functional impact scores (FISs) of genes are calculated on the basis of mutation data from Mutation Annotation Format (MAF) file and the FISs of mutations from MutationAssessor. Then, the background distribution of FISs is estimated by using the artificial neural network and hierarchical clustering algorithm with multi-omics features as input. Based on these, driver genes are identified by comparing the observed FIS to the background distribution.

The outline of FI-net includes (1) calculating the observed FISs for genes on the basis of Mutation Annotation Format (MAF) files and MutationAssessor (Reva et al., 2011), (2) building the artificial neural network to estimate the FISs for genes based on multi-omics features and estimating the background distribution of functional impact score in each cluster obtained by the hierarchical clustering algorithm, and (3) identifying driver genes by comparing the observed FIS to the background distribution in each cluster. The workflow of FI-net is shown in Figure 1.

### 2.1. Data

#### 2.1.1. Cancer Mutation Data

We used the MAF files from TCGA (available at https://tcga-data.nci.nih.gov/tcga/) to do the driver gene analysis. For each mutation in the MAF file, Hugo_Symbol, Chromosome, Start_Position, End_Position, Variant_Classification, Reference_Allele, Tumor_Seq_Allele, and Tumor_Sample_Barcode are essential information for analysis.

#### 2.1.2. Functional Impact Score of Mutation

FI-net used the FISs from MutationAssessor (Reva et al., 2011), which assessed the functional impacts of mutations based on evolutionary conservation of the affected amino acid in protein homologs. Significant score in MutationAssessor indicates the more likely functional impact of a mutation. The release 3 “MA scores rel3 hg19 full” (available at http://mutationassessor.org/r3/), containing the FISs for mutations in hg19 reference genome (chromosome 1–22, X, and Y), were adopted. Note that other methods evaluating the functional impacts of mutations [e.g., SIFT (Ng and Henikoff, 2003), GERP (Cooper et al., 2005), PolyPhen (Adzhubei et al., 2010), and CADD (Kircher et al., 2014)] can also be used in FI-net. The overlap analysis for the driver genes identified by FI-net using MutationAssessor and CADD has been summarized in Supplementary Material.

#### 2.1.3. Genetic Features From Multi-Omics Data Sources

Twelve genetic features from multi-omics data sources (genomics, transcriptomics, and epigenomics) were adopted to build an ANN model, including

1. the expression level from Lawrence et al. (2013);
2. the DNA replication timing from Lawrence et al. (2013);
3. the chromatin compartment (HiC) from Lawrence et al. (2013);
4. the length of genomic regions from Jiang et al. (2019);
5. the constraint score for non-synonymous mutations from Samocha et al. (2014);
6. the hubness in a gene expression network from Lee et al. (2018);
7. the gene's known regulatory role based on gene annotation databases from Lee et al. (2018)
8. the genomic copy number variation (CNA) from Lee et al. (2018);
9. the methylation status from Lee et al. (2018);
10. the total mutation number among patients calculated by local MAF file;
11. the deleterious mutation (including mutations with null and non-silent effects) number among patients calculated by local MAF file;
12. the standard deviation of functional impact score across patients calculated by local MAF file.

Some genes missed the feature values, such as the expression level and DNA replication timing. We proposed a method to compensate the missing values as follows:

1. Let $D_{i,j}=\sqrt{\underset{l\notin \left(S_{i}\cup S_{j}\right)}{\sum }{\left(v_{l,i}-v_{l,j}\right)}^{2}}$ denotes the distance between gene *i* and *j*, where *S*_*i*_ (*S*_*j*_) is the set of features which are missing in gene *i* (*j*), and *v*_*l, i*_ (*v*_*l, j*_) is the feature *l* of gene *i* (*j*). Let *N*_*g*_*k*__ denotes the set of the *K* nearest neighbor genes without missing value in feature *k* surrounding gene *g*. *K* was set to 100 in this research.
2. The missing feature *k* of gene *g*, $v_{k,g}^{*}$ was compensated by:

(1) $${\hat{v}}_{k,g}^{*}=\frac{1}{K}\sum_{t\in N_{g_{k}}}v_{k,t}$$

3. Each feature was centered and normalized as follows:

(2) $$z_{k,g}=\frac{v_{k,g}-\frac{1}{G}\sum_{i=1}^{G}v_{k,i}}{\sqrt{\frac{1}{G-1}\sum_{j=1}^{G}{\left(v_{k,j}-\frac{1}{G}\sum_{i=1}^{G}v_{k,i}\right)}^{2}}}$$

where *G* is the total number of genes under study.

### 2.2. Calculation of the Observed FISs for Genes

The calculation of the observed FISs for genes was divided into the following three steps:

1. Obtaining the FISs from MutationAssessor (Reva et al., 2011). The mutations in the MAF file were mapped to the mutations in “MA scores rel3 hg19 full” file according to the information of the loci of mutations and the reference-alteration bases.
2. Compensating the missing FISs: Some FISs of mutations cannot be evaluated by MutationAssessor. To this end, the variant classifications (such as silent, synonymous, nonsense, non-stop, and in-frame deletion) of mutations in the MAF file were mapped to the corresponding mutation effects (silent, non-silent, non-coding, and null) according to the “mutation type dictionary file” from Lawrence et al. (2013). Let *Q*_*j*_ denotes the set of mutations with effect *j* of which FISs are known. The missing FIS of mutation *i* with effect *j* was compensated by the average FIS of mutations with effect *j* as follows:

(3) $$f_{i,j}^{miss}=\frac{1}{\left|Q_{j}\right|}\sum_{k\in Q_{j}}f_{k,j}$$

where |*Q*_*j*_| is the cardinality of *Q*_*j*_ and *f*_*k, j*_ is the FIS of mutation *k* with effect *j*.
 Note that methods evaluating the functional impacts of mutations are always focused on the non-synonymous somatic mutations, such as MutationAssessor (Reva et al., 2011), SIFT (Ng and Henikoff, 2003), and PolyPhen (Adzhubei et al., 2010). The FISs of synonymous and some protein-affecting mutations, such as nonsense mutations and small indels, may be missing, and the average FIS of mutations with silent and null effect cannot be calculated from MutationAssessor. In general, the impact of silent, non-coding, non-silent, and null mutations on protein increases gradually. Silent mutations do not affect the amino acids of protein sequence, and they should be assigned the smallest FIS. Non-coding mutations do not alter amino acids, but they can promote tumor progression. For example, 3′-untranslated regions (3′UTR) non-coding mutations can alter microRNA (miRNA) binding efficiency and consequently trigger loss/gain of gene function (Akdeli et al., 2014; Wu et al., 2018). Non-silent mutations, which alter the amino acids of protein, may have significant functional impacts on protein and accelerate the progression of tumors. For example, R132 mutation in *IDH1* was found to be associated with early gliomagenesis (Yip et al., 2012). Null mutations including “nonsense mutation,” “splice-site,” “frameshift deletion,” “frameshift insertion,” and so on can cause continuous changes in amino acid sequence and have more significant impacts on the organism. Based on the above analysis, under the condition of the average FIS of effect *j* cannot be calculated, the FIS of mutation *i* with effect *j* was set to:

(4) $$f_{i,j}^{miss}=\{\begin{array}{ll} 0 & \text{mutation effect }j\text{ is silent}\\ 1 & \text{mutation effect }j\text{ is non-coding}\\ 2 & \text{mutation effect }j\text{ is non-silent}\\ 3 & \text{mutation effect }j\text{ is null} \end{array}$$

3. Calculating the observed FISs for genes. The observed FIS of gene *g* was calculated by:

(5) $$F_{g}^{obs}=\sum_{i=1}^{M_{g}}f_{i}^{g}$$

where *M*_*g*_ is the number of mutations in gene *g* and $f_{i}^{g}$ is the FIS of mutation *i* in gene *g*.

### 2.3. Estimation of the Background Distribution

#### 2.3.1. Artificial Neural Network Model

**Figure 2:**
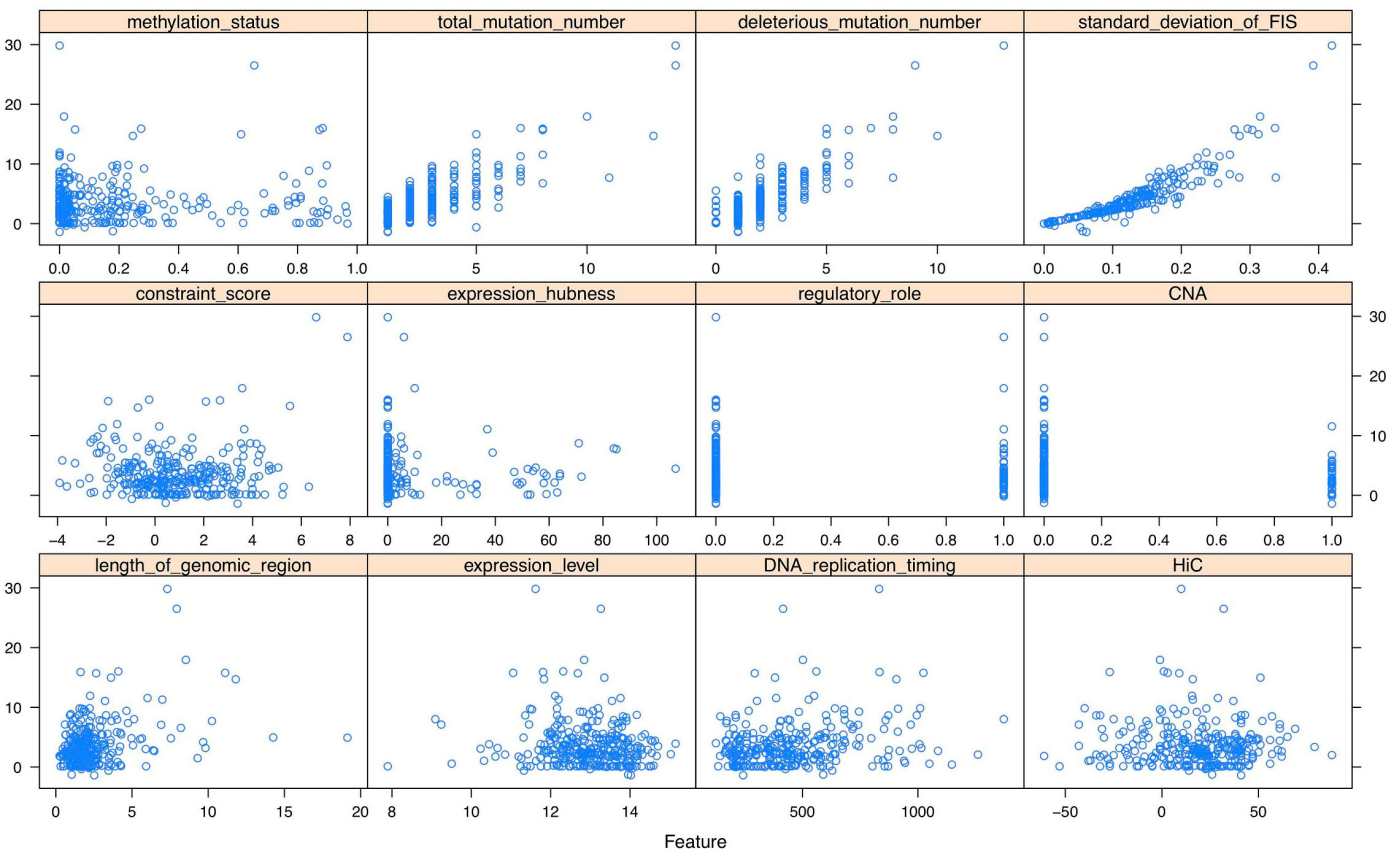
The scatter plots between functional impact score (FIS) and multi-omics features of 300 genes (randomly sampling) in breast invasive carcinoma (BRCA).

As shown in the scatter plots of Figure 2, Supplementary Figures 7, 8, there are non-linear relationships between FIS and the multi-omics features. Besides, we reduced the multi-omics features to 2-dimensional features using t-SNE method (Laurens and Hinton, 2008). The scatter plots in 3D space of Supplementary Figures 9–11 show that FISs and the features after dimensionality reduction also have non-linear relationships. Consequently, a feed-forward single hidden layer ANN was used to build a non-linear regression model on FIS by incorporating multi-omics features. Our network architecture consists of three layers of interconnected neuron units, including the input layer, the single hidden layer of non-linearity, and the output layer. The multi-omics feature matrix $Z={\left(z_{1},z_{2},\ldots ,z_{G}\right)}^{T}\in {\mathbb{R}}^{G\times p}$ was used as the multivariate input, with *G* being the number of genes and *p* = 12 being the number of multi-omics features. The feature vector $z_{g}={\left(z_{1,g},z_{2,g},\cdots \;,z_{12,g}\right)}^{T}$ for *g* = 1, 2, …, *G* was passed through the three layers according to:

(6) $$\begin{array}{l} \text{Input layer}:u^{\left(1\right)}=y^{\left(1\right)}=z_{g}\\ \text{Hidden layer}:\{\begin{array}{l} u^{\left(l+1\right)}=\;W^{\left(l+1\right)}y^{\left(l\right)}+b^{\left(l+1\right)}\\ y^{\left(l+1\right)}=f\left(u^{\left(l+1\right)}\right) \end{array}\left(l=1,2\right)\\ \text{Output layer}:{\hat{F}}_{g}=y^{\left(3\right)} \end{array}$$

where *u*^(*l*)^ is the input of layer *l*, *y*^(*l*)^ is the output of layer *l*, and ${\hat{F}}_{g}$ is the estimated FIS of gene *g*. The parameters *W*^(2)^, *b*^(2)^, *W*^(3)^, and *b*^(3)^ were trained by the back-propagation algorithm. The single hidden layer contains 100 neurons, then *W*^(2)^ ∈ ℝ^100×12^, *b*^(2)^ ∈ ℝ^100×1^, *W*^(3)^ ∈ ℝ^1×100^, and *b*^(3)^ ∈ ℝ. ReLU function, *f*(*x*) = max(0, *x*), was used as the non-linear activation function. R package h2o (http://h2o.ai/resources) has been used to construct and set up the ANN model in this study. The number of training epochs is 10.

#### 2.3.2. The Distribution of Background Functional Impact Score

Hierarchical clustering algorithm has been proven effective across a range of applications, including genomic data analysis (Aceto et al., 2014; Pagnuco et al., 2017; Won et al., 2020). For estimating the local distribution of BFIS, we implemented the hierarchical clustering algorithm to group genes with similar multi-omics features together. Ward's method (Murtagh and Legendre, 2014), which is based on an error sum of squares criterion, was used in the hierarchical clustering. It produced clusters by minimizing the within-group dispersion at each binary fusion. The Euclidean distance was used to measure the distance between genes *i* and *j* as follows:

(7) $$D_{i,j}={\left\|z_{i}-z_{j}\right\|}_{2}^{2}$$

**Figure 3:**
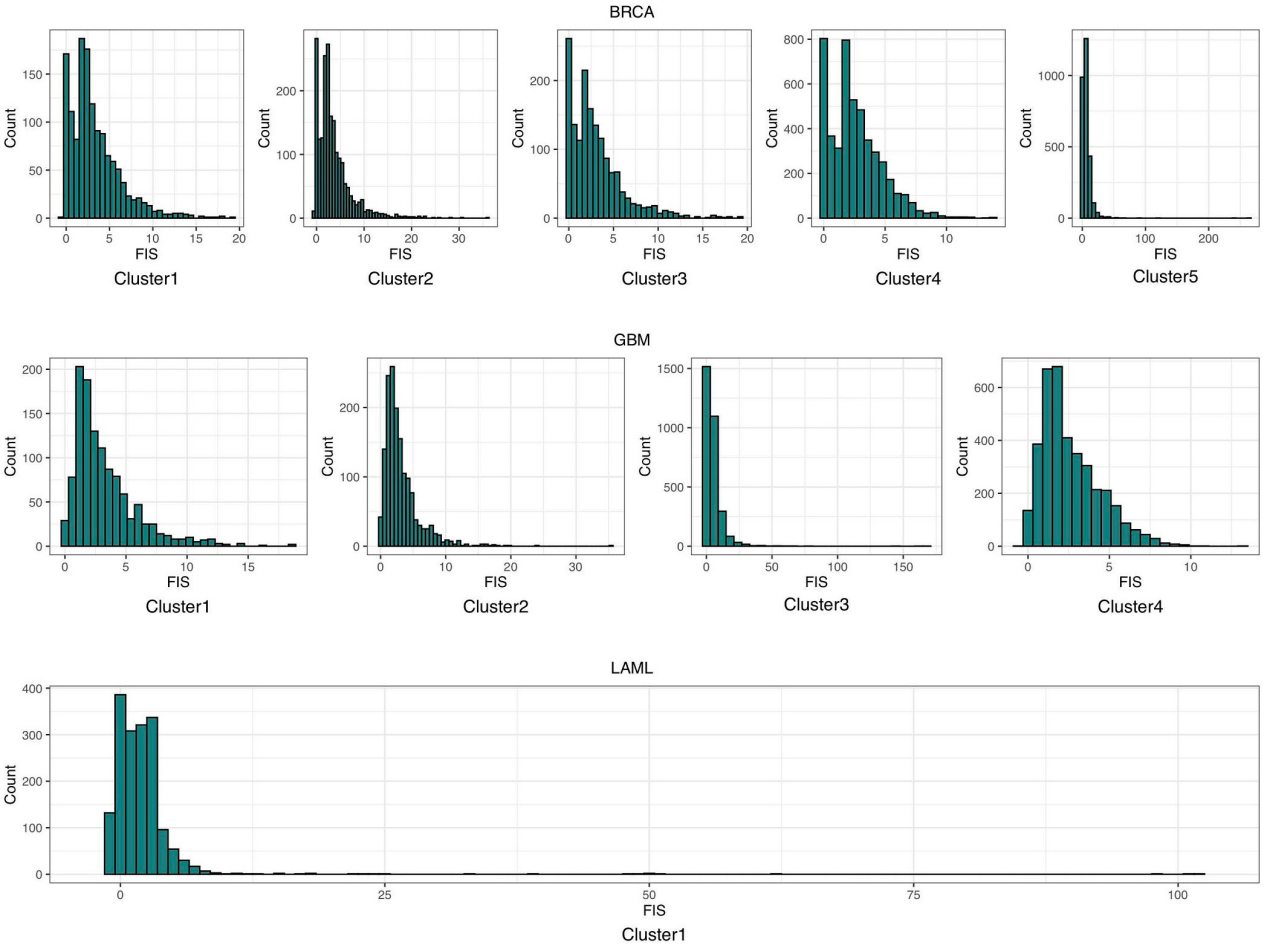
The histograms of the estimated functional impact scores (FISs) in breast invasive carcinoma (BRCA), glioblastoma multiforme (GBM), and acute myeloid leukemia (LAML).

As shown in the histograms in Figure 3, most of the distributions of estimated FISs in each cluster can be approximated by the gamma distribution. Thus, a gamma distribution was fitted for getting the local distribution of BFIS. The number of clusters influences the background distribution, and hence affects the performance of identifying driver genes. The number of clusters *N*_*c*_ was set as follows:

(8) $$N_{c}=\left\lceil \frac{G}{N}\right\rceil$$

where *G* is the total number of genes under study, *N* is a predefined expected number of genes in each cluster. The performance of FI-net for different values of *N* (1,000, 2,000, 3,000, 4,000, and 5,000) is shown in Supplementary Material. The number of identified driver genes increases as *N* increases, and the proportion of overlap with the CGC driver list decreases as *N* increases. In this study, *N*= 3,000.

As gamma distribution is a positively skewed distribution, we removed the outliers with minor estimated FISs. To this end, 5%-truncated estimated FISs instead of the entire data were used to fit the distribution. In detail, estimated FISs below 5% quantile in each cluster were removed. For clusters with non-positive FISs, an overall adjustment was performed to guarantee that all FISs are within the domain of gamma distribution. The estimated FIS of gene *g* in cluster *c* with non-positive FISs was adjusted by:

(9) $${\hat{F}}_{g}\leftarrow {\hat{F}}_{g}-\underset{g\in S_{c}}{\min}{\hat{F}}_{g}+0.01$$

where $\underset{g\in S_{c}}{\min}{\hat{F}}_{g}$ is the minimum FIS in cluster *c* and *S*_*c*_ is the set of genes in cluster *c*.

The shape parameter α_*c*_ and the scale parameter β_*c*_ of gamma distribution in cluster *c* were estimated by maximizing the following likelihood function:

(10) $$L({\hat{\alpha }}_{c},{\hat{\beta }}_{c})=\prod_{g=1}^{G_{c}}f({\hat{F}}_{g}|{\hat{\alpha }}_{c},{\hat{\beta }}_{c})$$

with

(11) $$f({\hat{F}}_{g}|{\hat{\alpha }}_{c},{\hat{\beta }}_{c})=\frac{{\hat{\beta }}_{c}^{{\hat{\alpha }}_{c}}}{\Gamma ({\hat{\alpha }}_{c})}{\hat{F}}_{g}{}^{{\hat{\alpha }}_{c}-1}\exp\left(-{\hat{\beta }}_{c}{\hat{F}}_{i}\right)$$

being the density function of the gamma distribution. *G*_*c*_ is the number of genes in cluster *c*. $\Gamma \left({\hat{\alpha }}_{c}\right)=\int_{0}^{\infty }e^{-x}x^{{\hat{\alpha }}_{c}-1}dx\left({\hat{\alpha }}_{c}>0\right)$.

### 2.4. Identification of Driver Genes

The identification of driver genes was performed in each cluster. The *p*-values of genes with significantly low FISs (≤0) were set to 1. Otherwise, to test the significance level of genes in cluster *c*, the null hypothesis was set up as follows: the observed FIS of gene *i* was assumed to obey the gamma distribution with parameters $\left({\hat{\alpha }}_{c},{\hat{\beta }}_{c}\right)$ estimated in section 2.3.2. The *p*-value of gene *g* was given by:

(12) $$p_{g}=1-H_{C}(F_{g}^{obs},{\hat{\alpha }}_{c},{\hat{\beta }}_{c})$$

with

(13) $$H_{C}=\int_{0}^{F_{g}^{obs}}\frac{{\hat{\beta }}_{c}^{{\hat{\alpha }}_{c}}}{\Gamma ({\hat{\alpha }}_{c})}x^{{\hat{\alpha }}_{c}-1}\exp\left(-{\hat{\beta }}_{c}x\right)dx$$

being the cumulative function of the gamma distribution.

The Benjamini–Hochberg false discovery rate algorithm was further applied to assign a *q*-value for each gene. In each cluster, genes exceeding the significance threshold (*q*-value ≤ 0.05) were identified as driver genes. Finally, the identified genes in all of the clusters were reported as driver genes by FI-net.

## 3. Results

We applied FI-net to the 31 datasets from the TCGA project, which have been summarized in DriverML (Han et al., 2019) and DriverDBv2 (Chung et al., 2016). FI-net was compared with other 22 associated methods, including NetBox (Cerami et al., 2010), Simon (Youn and Simon, 2011), Dendrix (Vandin et al., 2012), MDPFinder (Zhao et al., 2012), MEMo (Ciriello et al., 2012), DriverNet (Bashashati et al., 2012), OncodriverFM (Gonzalezperez and Lopezbigas, 2012), ActiveDriver (Reimand and Bader, 2013), DrGaP (Hua et al., 2013), iPAC (Ryslik et al., 2013), MutSigCV (Lawrence et al., 2013), OncodriveCLUST (Tamborero et al., 2013a), CoMDP (Zhang et al., 2014), DawnRank (Hou and Ma, 2014), e-Driver (Portapardo and Godzik, 2014), MSEA (Jia et al., 2014), OncodriveFML (Mularoni et al., 2016), ExInAtor (Lanzos et al., 2017), rDriver (Wang et al., 2018), SCS (Guo et al., 2018), DriverML (Han et al., 2019), and UniCovEx (Gao et al., 2019). The driver gene lists of the first 21 methods were obtained from DriverML and DriverDBv2. UniCovEx was run using the default parameters, and all genes in MAF files were taken as considered genes. Gene modules [only the 50 modules with the highest comprehensive score in each protein–protein interaction (PPI) network were considered] output by at least 2 of the 3 PPI networks (HINT + HI2012, iRefIndex, and Multinet) were selected as the final predictions. All genes in the predicted gene modules were identified as driver genes.

### 3.1. FI-Net Identifies the Adequate Number of Driver Genes

**Figure 4:**
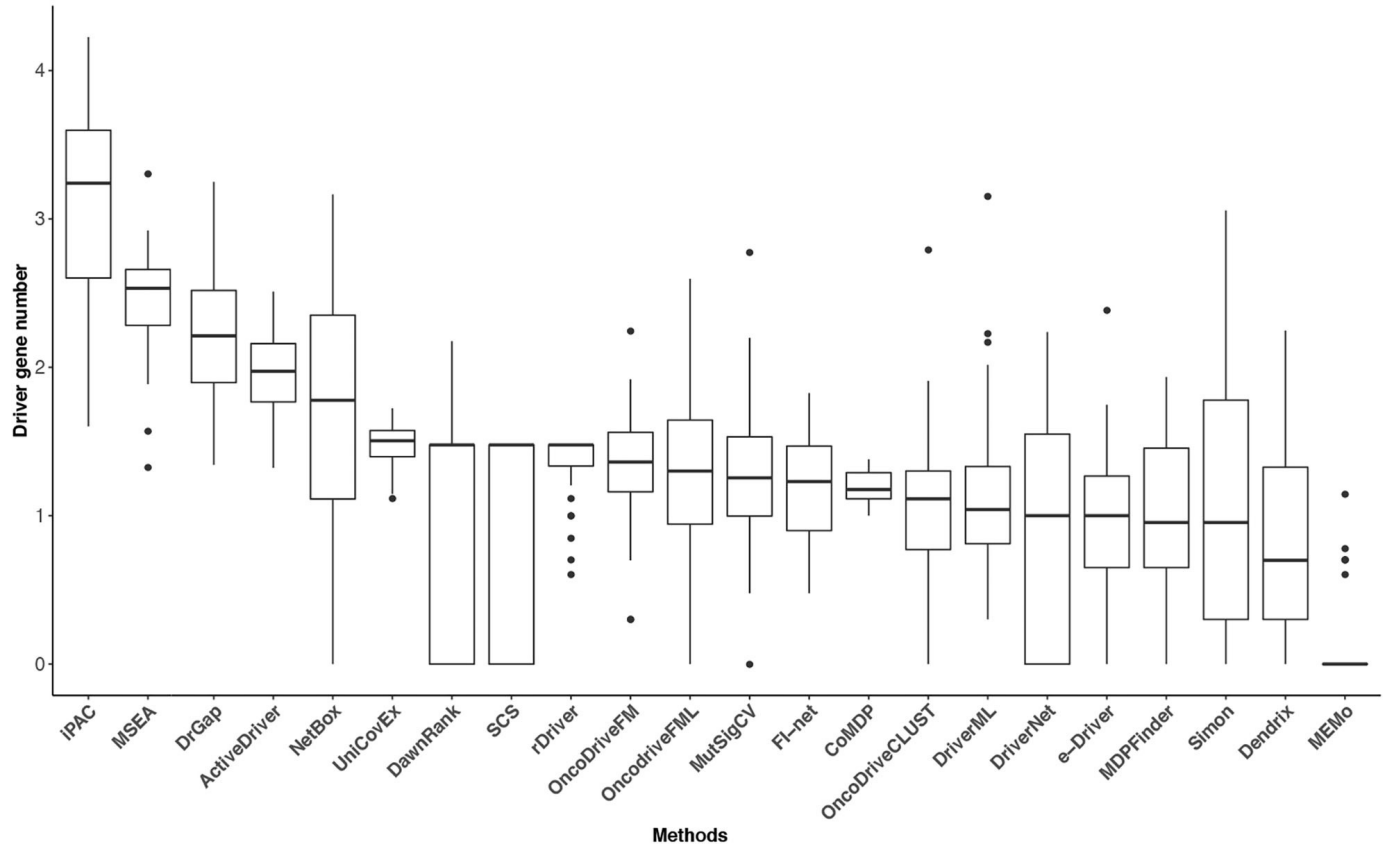
The driver gene number (on a log-10 scale) of the 22 methods in the 31 TCGA datasets. The numbers of driver genes predicted by different methods differ significantly.

Tumor heterogeneity is widespread, and the mutation frequency and driver genes across patients with a given type of tumor are various (Vandin et al., 2012; Lawrence et al., 2013). The tumor heterogeneity inflates the number of putative driver genes, and the number of driver genes may have some variability among cancer types. However, the classic epidemiologic studies and sequencing data analysis have suggested that a typical tumor ordinarily contains 2–8 driver gene mutations, and the remaining gene mutations are passengers that show no selective growth advantage for tumor (Armitage and Doll, 1954; Vogelstein et al., 2013). Driver gene identification methods based on sequencing data analysis is to reduce the scope of research for biological experiment methods. It is crucial to obtain an adequate number of driver genes for these methods. Too few identified driver genes may miss some critical cancer targets, and too many genes will cause difficulties for subsequent biological verification and further studies. The numbers of identified driver genes across the 31 datasets were summarized in Figure 4. The median of driver genes among the 31 datasets ranged from 0 (MEMo) to 1,740 (iPAC). Several methods exhibited underselection, which means they detect too few driver genes. MEMo, SCS, DawnRank, DriverNet, Simon, OncoDriveCLUST, NetBox, e-Driver, and MutSigCV identified no driver genes in some datasets. The interquartile range (IQR) of the numbers of driver genes identified by some methods was huge. For instance, iPAC detected 40 driver genes in acute myeloid leukemia (LAML) and 16,799 driver genes in liver hepatocellular carcinoma with IQR being 3,633. The number of driver genes identified by FI-net in the 31 datasets ranged from 3 to 67 with median being 17. The driver genes predicted by FI-net have been summarized in Supplementary Table 1. The IQR of the number of driver genes identified by FI-net was 21, with nine datasets obtaining fewer than 10 genes and eight datasets obtaining more than 30 genes.

### 3.2. FI-Net Is of Best Precision According to Overlaps With CGC and NCG Driver List

**Figure 5:**
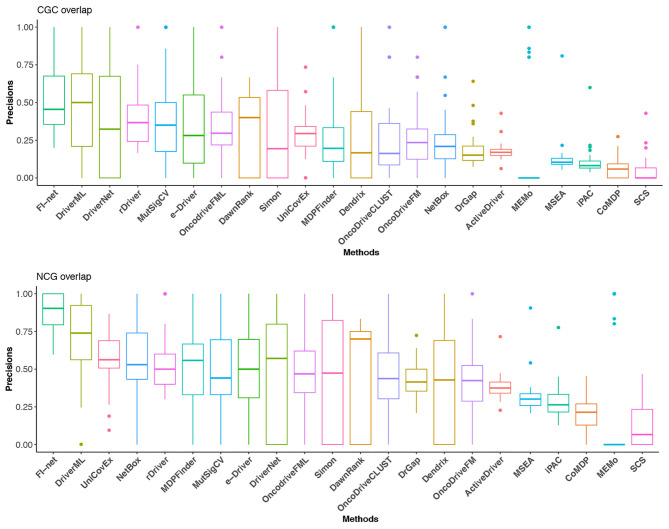
The precisions in CGC and NCG of the 22 methods in the 31 TCGA datasets. Methods are sorted with respect to the mean of precisions.

With respect to Portapardo and Godzik (2014), Lanzos et al. (2017), Wang et al. (2018, 2020), Gao et al. (2019), Guo et al. (2019), and Han et al. (2019), the overlaps with the gene lists from CGC and NCG database were used as criteria to evaluate the performance of methods. To this end, the proportion of overlap was denoted as the precision of a method. For the method that identified fewer than three driver genes, the precision was set to 0. The precisions in CGC and NCG of 23 methods in the 31 TCGA datasets are illustrated in Figure 5 and Supplementary Tables 2, 3. In the boxplot of Figure 5, the methods were sorted for the mean of precisions. The top three methods in CGC database were FI-net, DriverML, and DriverNet with the average precision among 31 datasets being 53.01, 48.19, and 39.38%, respectively. FI-net achieved a precision >50% in 14 of the 31 datasets. Therein, the precisions in brain lower grade glioma and uveal melanoma were 100%. The top three methods in NCG database were FI-net, DriverML, and UniCovEx with the average precision being 88.20, 70.55, and 55.70%. All driver genes identified by FI-net in eight datasets are in NCG database.

### 3.3. FI-Net Identifies Driver Genes With High Deleterious Mutation Ratio and High Coverage

**Figure 6:**
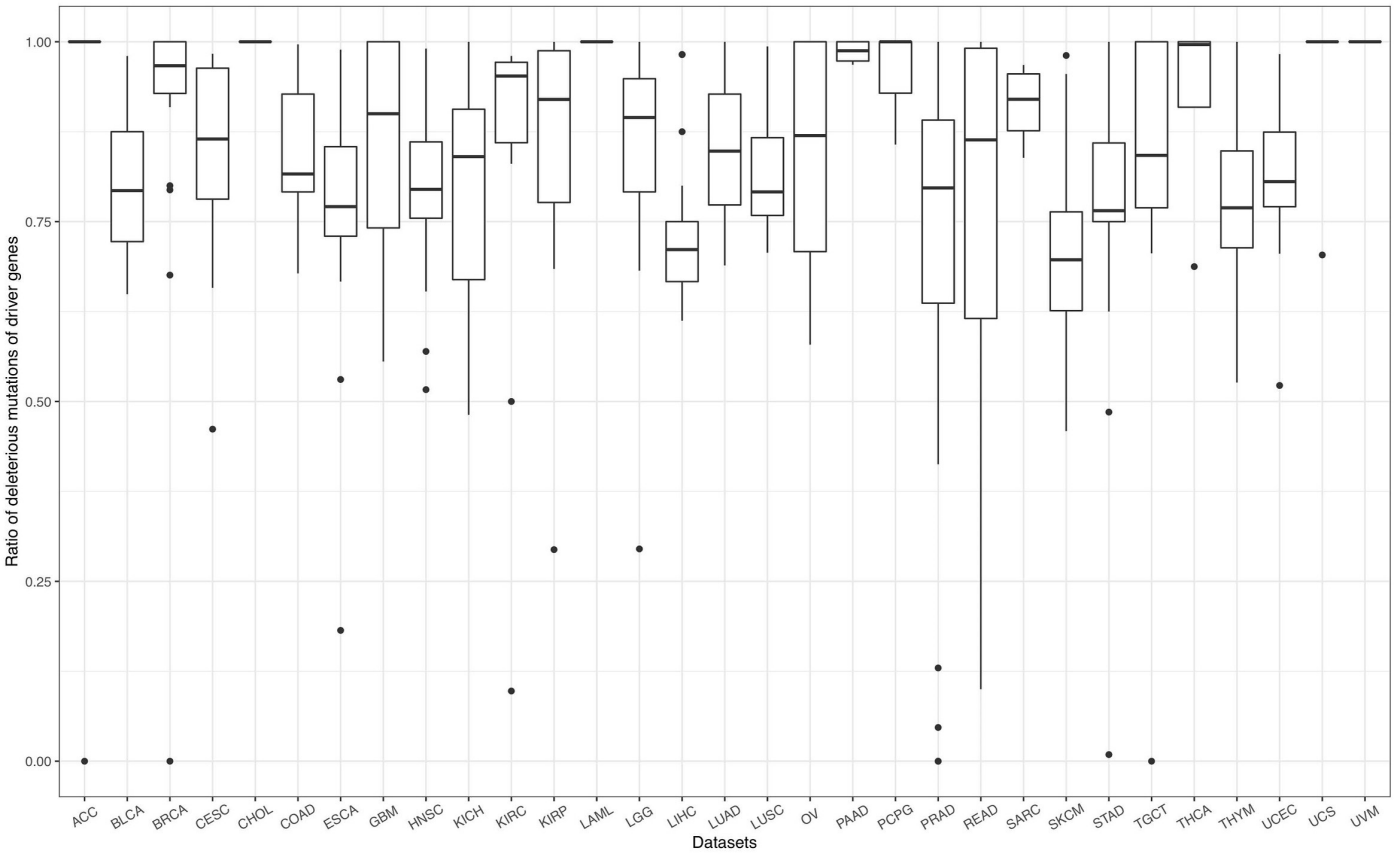
The ratio of non-silent and null mutations (deleterious mutations) to total mutations for driver genes identified by FI-net. Thirty-one boxplots show the ratios of deleterious mutations in all genes identified in 31 datasets, respectively.

As mentioned in section 2.2, mutations can be classified into four effects, including silent, non-silent, non-coding, and null. Therein, silent mutations (synonymous mutations) in the gene coding sequence, and non-coding mutations in the flanking untranslated regions (UTR) and intronic sequences, safely beyond functional splice site mutations show weak selective growth advantage for tumor and can be considered as background mutations (Lawrence et al., 2013). Non-silent and null, which will affect the amino acids of protein or even cause frameshift of the sequence, play major roles in tumorigenesis. Based on a long history in the study of selection in species evolution, Martincorena et al. (2017) proposed an index, dN/dS, the normalized ratio of non-synonymous to synonymous mutations to quantify selection in cancer genomes. They demonstrated that genes, which show significant high dN/dS ratio, tend to show positive selection in tumor cells. The similar idea can also be seen in Lawrence et al. (2013) and Tokheim et al. (2016). Tokheim et al. (2016) used a ratiometric feature, the median ratio of non-silent to silent mutations, to evaluate the performance of driver gene prediction methods. Driver genes identified by Lawrence et al. (2013) showed high ratios of the protein-affecting mutations to other mutations. Inspired by these studies, we defined a ratiometric feature, the ratio of non-silent and null mutations (deleterious mutations) to total mutations in each gene. The ratios of deleterious mutations for driver genes identified by FI-net have been summarized in Figure 6 and Supplementary Table 4. The average ratio of driver genes identified by FI-net among 31 datasets is 0.8455. Eighty-five in 609 driver genes were with ratio being 1, that is, all mutations in these 85 genes are non-silent or null mutations.

**Figure 7:**
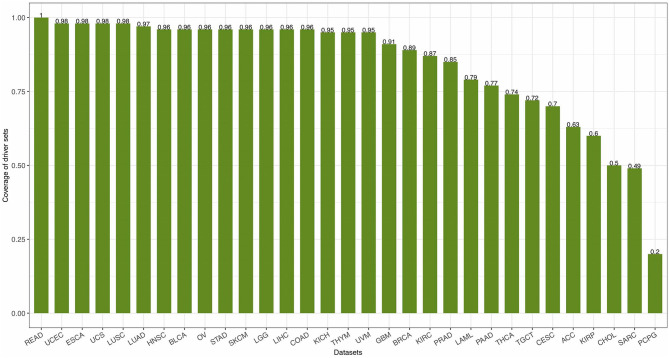
The coverage of driver gene sets identified by functional impact prediction neural network (FI-net) in the 31 datasets.

For each cancer dataset, all driver genes identified from a cohort of patients with a given type of tumor can be seen as a driver gene set. Driver gene set tends to show high coverage, which means mutations in members of the driver gene set recurrently occur in patient cohorts (Vandin et al., 2012; Xu et al., 2019). The coverage of gene set *S* is the proportion of patients with mutations in the genes of *S* to all patients under study and can be defined as:

(14) $${\text{Cov}}_{S}=\frac{1}{m}|P_{S}|$$

with *P*_*S*_ being the set of patients with mutations in the genes of *S* and *m* being the total number of patients. The coverage of 31 driver gene sets [calculated by Equation (14)] identified by FI-net in the 31 datasets ranged from 0.2011 to 1.00 with average coverage being 0.8419 (Figure 7).

### 3.4. FI-Net Identifies Known and Potential Novel Driver Genes

Genes identified by multiple tools simultaneously may be driver genes, because the false positives of one method may be thrown away by other methods (Tamborero et al., 2013a). A total of 609 driver genes were identified by FI-net in the 31 datasets, and 576 of them were also detected by other methods. All driver genes identified by FI-net in 15 of 31 datasets were also detected by other methods. For example, FI-net identified known driver genes, *DNMT3A, FLT3, NPM1, IDH1, IDH2, TET2, TP53, RUNX1, CEBPA, NRAS, WT1, U2AF1*, and *KRAS*, in LAML, which were detected by at least 10 other methods and presented in CGC and NCG database. FI-net identified *PIK3CA, TP53, MAP3K1, GATA3, KMT2C, CDH1, RUNX1, BRCA1, BRCA2, FAT3, MAP2K4, PTEN, ATM, CROCCP2, USH2A, RYR2, HMCN1, NEB*, and *FLG* in BRCA. The first 13 genes were presented in CGC database. All genes except *CROCCP2* were predicted by at least two other methods and presented in NCG database. The overlap between FI-net and other three newest methods OncodriveFML (functional-based method), DriverML (frequency-based method), and UniCovEx (Gao et al., 2019) (pathway-based method) in their predictions of LAML and BRCA are shown in Supplementary Figures 19, 20.

According to Hou and Ma (2014), Portapardo and Godzik (2014), and Han et al. (2019), the potential novel driver genes always show the following properties:

1. they have not been detected by the driver gene detection methods;
2. they were not presented in the CGC database;
3. they were supported to be related to the development of cancers by convincing studies.

Although the driver genes identified by FI-net in ovarian serous cystadenocarcinoma, prostate adenocarcinoma, and adrenocortical carcinoma got the worst performance in terms of the consensus with other methods. Note that 77.78, 82.35, and 83.33% of driver genes in these 3 datasets were also detected by other methods. In ovarian serous cystadenocarcinoma, FI-net identified *TP53, NF1, BRCA1, BRCA2, MUC16, CSMD3, FAT3, EGFR, RB1, CDK12, HMCN1, USH2A, CACNA1C, DST, MUC17, DNAH5, LRP2, RYR2, PRKDC, SON, GPR98, ZFYVE26, AHNAK2, GLI2, APOB, ZNF236*, and *ODZ1*. Therein, the first 10 genes presented in the CGC database. The first 20 genes were also detected by other methods. For the remaining genes, *GLI2* increases abnormally in benign tumors and ovarian cancer tissues (Zhang et al., 2019), and regulates the survivin isoform expression in ovarian cancer (Trnski et al., 2019). The other 6 genes, *GPR98, ZFYVE26, AHNAK2, APOB, ZNF236*, and *ODZ1*, were all supported to be related to cancers by research (Hatano et al., 2008; Sagona et al., 2011; Backes et al., 2015; Borgquist et al., 2016; Lu et al., 2017; Talamillo et al., 2017). Even though these seven newly identified genes were not known driver genes, they met the three properties of novel driver genes.

### 3.5. Overall Performance

The efficiencies of 23 tools have been evaluated based on the overlap fraction with CGC, NCG, and the consensus in prediction of driver genes among methods. Genes identified by several methods simultaneously can be considered as critical driver genes (Tamborero et al., 2013b). Method consensus shows the ability to identify the genes that are also identified as potential driver genes by many other methods. For each method, we calculated the fraction of predicted driver genes that were predicted by at least one other method denoted as “consensus No. 1” and by half of 23 methods (11 methods) denoted as “consensus No. 2.” To measure the overall performance of methods clearly, we summarized the average value among 31 datasets for these four criteria in Table 1. Each method is accordingly ranked by these 4 criteria and the average rank is shown. In cholangiocarcinoma and kidney renal papillary cell carcinoma datasets, there are no common driver genes that were identified by half of the methods. The average consensus value among the remaining 29 datasets was calculated for consensus No. 2. In summary, the top-ranked three methods are FI-net, DriverML, and MutSigCV with the average rank being 1.75, 2.75, and 5.75.

**Table 1:** Overall performances of 22 driver gene prediction methods on 31 TCGA datasets.

**Methods**	**CGC overlap**	**NCG overlap**	**Consensus No.1**	**Consensus No.2**	**CGC rank**	**NCG rank**	**Consensus No.1 rank**	**Consensus No.2 rank**	**Average rank**
ActiveDriver	17.92%	38.51%	52.28%	2.03%	17	17	18	20	18
Dendrix	28.75%	42.26%	69.38%	19.11%	12	15	12	6	11.25
MDPFinder	28.82%	51.58%	79.34%	24.15%	11	6	8	2	6.75
Simon	29.25%	45.26%	62.13%	7.36%	9	11	14	14	12.25
NetBox	26.41%	54.26%	74.18%	11.10%	15	4	11	13	10.75
OncoDriveFM	26.52%	42.04%	76.92%	13.96%	14	16	10	9	12.25
MutSigCV	37.07%	51.30%	89.94%	18.24%	5	7	3	8	5.75
MEMo	17.07%	18.17%	18.71%	11.37%	18	22	23	11	18.5
CoMDP	6.70%	20.39%	37.89%	0.51%	22	21	19	22	21
DawnRank	31.66%	44.97%	36.60%	3.08%	8	12	20	18	14.5
DriverNet	39.38%	50.67%	59.15%	22.39%	3	9	16	3	7.75
e-Driver	36.07%	51.05%	78.85%	**28.65%**	6	8	9	**1**	6
iPAC	11.13%	29.16%	32.71%	1.38%	21	20	21	21	20.75
MSEA	13.36%	32.01%	64.81%	2.58%	20	19	14	19	18
OncoDriveCLUST	44.32%	21.61%	87.10%	19.38%	13	13	6	7	9.75
DrGap	18.81%	42.69%	88.79%	3.30%	16	14	4	16	12.5
DriverML	48.19%	70.55%	94.01%	20.38%	2	2	2	5	2.75
OncodriveFML	33.78%	48.03%	81.15%	11.02%	7	10	7	12	9
SCS	5.15%	1.32%	19.66%	0.23%	23	23	22	23	22.75
rDriver	38.18%	53.17%	87.89%	12.97%	4	5	5	10	6
UniCovEx	29.01%	55.70%	65.56%	3.52%	10	3	13	17	10.75
FI-net	**53.01%**	**88.20%**	**95.18%**	21.46%	**1**	**1**	**1**	4	**1.75**

*The bold number indicates the best result*.

## 4. Discussion

A key task in cancer genomics research is to identify driver genes that contribute to the progression of cancer (Han et al., 2019). The protein-affecting mutations in certain gene regions, which reflects the functional impacts of genes, tend to be targeted in the tumorigenesis (Tamborero et al., 2013a). The potential driver genes show the bias toward the accumulation of the functional mutations, including non-synonymous, missense, and stop site mutations (Gonzalezperez and Lopezbigas, 2012; Tamborero et al., 2013a; Portapardo and Godzik, 2014). Motivated by these facts, the function-based methods were developed. However, the existing function-based methods always estimate the distribution of the null model using the non-parametric method, such as random sampling, which is limited by the sample size (Whitley and Ball, 2002). The background distribution estimated by non-parametric method could probably be biased for the datasets with small sizes. This estimation bias can increase the detection rate of false positives. Meanwhile, the underselection and overselection cannot be overlooked for driver gene detection methods. Nine of 23 methods identified no driver genes, and 7 of 23 methods detected thousands of driver genes in some datasets. The large variance of driver gene number might bring significant uncertainties for the further applications of these methods.

We proposed FI-net method to identify driver genes based on functional impact prediction neural network. The neural network model was widely used in the research of bioinformatics and achieved excellent performance (Dwivedi, 2018; Eetemadi and Tagkopoulos, 2019; Tsou and Wu, 2019). To tackle the shortcomings of non-parametric estimation in the function-based method, an ANN model with one single hidden layer was trained to learn the non-linear relationship between the FISs and the multi-omics features. Because ANN is a kind of parametric models, FI-net can be expected to be robust against the change of the data comparing with the non-parametric models. The multi-omics features, such as expression level and the DNA replication timing, have been reported to be correlated with the mutation frequencies (Lawrence et al., 2013). Thus, the assumption of the identical distribution of mutation frequencies for all the genes is not proper in estimating the background distribution.

FI-net was proposed by fully considering the multi-omics features of genes and the probabilistic characteristics of FISs. It is known that the genes with different multi-omics features are of various mechanisms. Thus, the identical probability distribution cannot be assumed for all the genes. To solve this problem, FI-net grouped genes with similar multi-omics features using Ward's clustering algorithm and identified driver genes in each cluster. The similar idea can be found in MutSigCV, which built bagels using three genetic features and estimated the background mutation rate within bagels. Because the distribution of FISs was approximately positive and skewed, we assumed that FISs in each cluster obey a gamma distribution. As a result, the non-significant genes can be properly filtered out.

We demonstrated that FI-net was of excellent performance by using the TCGA mutation data. FI-net was proved to overcome the problem of underselection and overselection and detect adequate number of driver genes. The number of driver genes in the 31 TCGA datasets varied from 3 to 67 with median being 17 and IQR being 21. For investigating the precision of detecting driver genes, FI-net was compared with other 22 associated methods on the percentage of overlaps with the CGC and NCG database. FI-net ranked first among 23 methods with average precision in CGC and NCG database being 53.01 and 88.20%. Furthermore, most of the driver genes identified by FI-net were of high deleterious mutation ratio and high coverage. The average deleterious mutation ratio of 609 driver genes was 0.8455. All mutations in 85 driver genes were considered to be deleterious mutations. The average coverage of 31 driver gene sets of FI-net was 0.8419.

Some limitations should be acknowledged in this research. First, we detected driver genes without considering the interaction between genes in the expression regulation networks. Genes known to regulate other genes or with many downstream genes are more likely to drive disease, including cancer (Lee et al., 2018). Some prior knowledge, such as the number of downstream genes, should be taken into consideration in future research. Second, driver genes were identified among a cohort of patients with the same type of tumor. In future research, the identification of patient-specific driver genes should be embedded for precision medicine. Last but not least, we directly used FISs of mutations from MutationAssessor, which made our method lack systematicness and integrity. Tools for evaluating the functional impacts of mutations always focus on the coding region, such as SIFT and PolyPhen. Future research will explore some advanced machine learning algorithms for predicting the functional impact of mutations in both coding and non-coding regions and integrate a user-friendly tool for identifying driver genes.

Our study first introduced and successfully applied the parametric model to estimate the distribution of BFIS by using multi-omics features. Another novelty of this research is estimating the background distribution and identifying driver genes within clusters obtained in the multi-omics feature space. Moreover, some false positives can be filtered by assuming the null distribution as a long-tailed gamma distribution. This study may provide a new perspective for the function-based methods.

## Data Availability Statement

Publicly available datasets were analyzed in this study. This data can be found at: https://tcga-data.nci.nih.gov/tcga/.

## Author Contributions

XX and PQ processed the data, designed the algorithm and the programming codes, and written the manuscript. HG and JW supervised the project and contributed to writing the manuscript. All authors contributed to the article and approved the submitted version.

## Conflict of Interest

The authors declare that the research was conducted in the absence of any commercial or financial relationships that could be construed as a potential conflict of interest.

## Supplementary Material

Supplementary Table 1 The driver genes predicted by FI-net.

Supplementary Table 2 The precisions in CGC of the 23 methods in the 31 TCGA datasets.

Supplementary Table 3 The precisions in NCG of the 23 methods in the 31 TCGA datasets.

Supplementary Table 4 The ratios of deleterious mutations for driver genes identified by FI-net.
