# Corrigendum: FI-Net: Identification of Cancer Driver Genes by Using Functional Impact Prediction Neural Network

**DOI:** 10.3389/fgene.2021.675351

**Published:** 2021-04-13

**Authors:** Hong Gu, Xiaolu Xu, Pan Qin, Jia Wang

**Affiliations:** ^1^Faculty of Electronic Information and Electrical Engineering, Dalian University of Technology, Dalian, China; ^2^Department of Breast Surgery, Institute of Breast Disease, Second Hospital of Dalian Medical University, Dalian, China

**Keywords:** cancer research, driver genes, functional impact, artificial neural network, multi-omics features, hierarchical clustering algorithm

In the original article, there was an error. The “ExInAtor” code was incorrectly used for a comparison with our method. ExInAtor is designed to only be used with mutations from Whole Genome Sequences, not with Whole Exome Sequences as was done in this article. As such, the ExInAtor results present in the Supplementary Material and those referenced in the text and Figures should be discounted.

A correction has been made to Figure 4. The corrected Figure 4 appears below.

**Figure 4:**
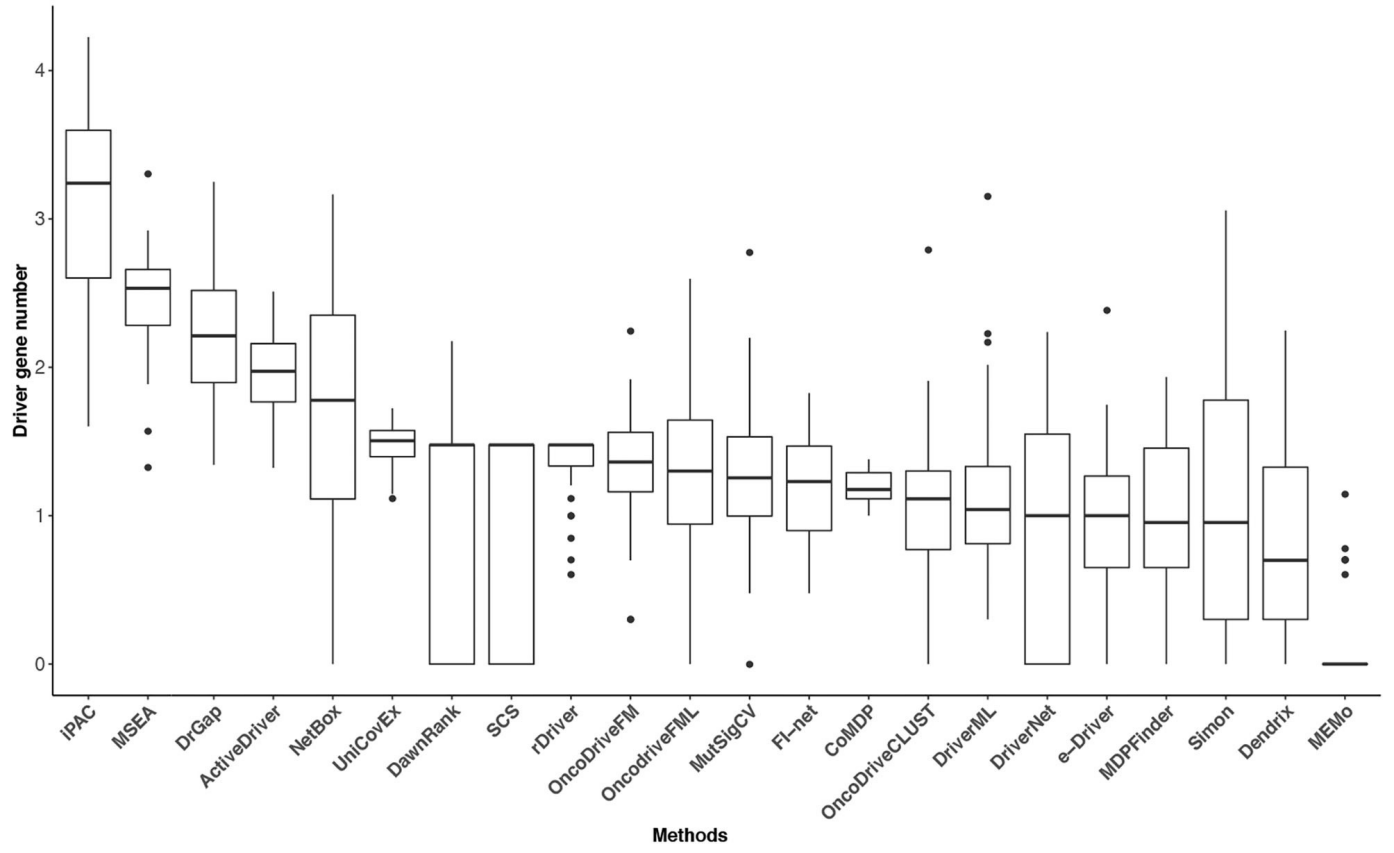
The driver gene number (on a log-10 scale) of the 22 methods in the 31 TCGA datasets. The numbers of driver genes predicted by different methods differ significantly.

Further, a correction has been made to Figure 5. The corrected Figure 5 appears below.

**Figure 5:**
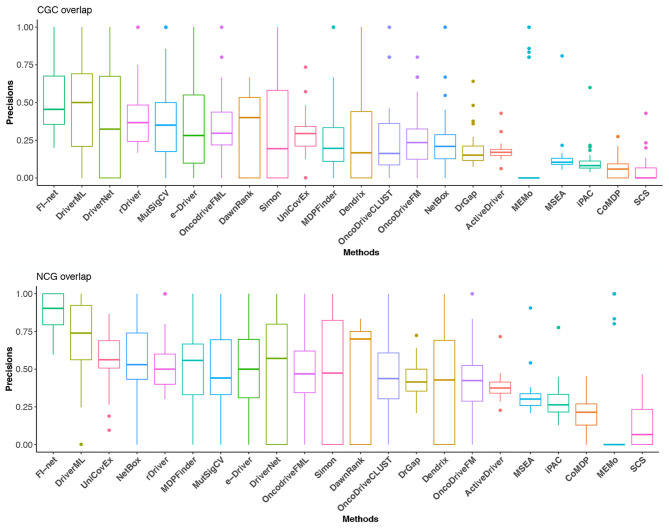
The precisions in CGC and NCG of the 22 methods in the 31 TCGA datasets. Methods are sorted with respect to the mean of precisions.

Lastly, a correction has been made to Table 1. The corrected Table 1 appears below.

**Table 1:** Overall performances of 22 driver gene prediction methods on 31 TCGA datasets.

**Methods**	**CGC overlap**	**NCG overlap**	**Consensus No.1**	**Consensus No.2**	**CGC rank**	**NCG rank**	**Consensus No.1 rank**	**Consensus No.2 rank**	**Average rank**
ActiveDriver	17.92%	38.51%	52.28%	2.03%	17	17	18	20	18
Dendrix	28.75%	42.26%	69.38%	19.11%	12	15	12	6	11.25
MDPFinder	28.82%	51.58%	79.34%	24.15%	11	6	8	2	6.75
Simon	29.25%	45.26%	62.13%	7.36%	9	11	14	14	12.25
NetBox	26.41%	54.26%	74.18%	11.10%	15	4	11	13	10.75
OncoDriveFM	26.52%	42.04%	76.92%	13.96%	14	16	10	9	12.25
MutSigCV	37.07%	51.30%	89.94%	18.24%	5	7	3	8	5.75
MEMo	17.07%	18.17%	18.71%	11.37%	18	22	23	11	18.5
CoMDP	6.70%	20.39%	37.89%	0.51%	22	21	19	22	21
DawnRank	31.66%	44.97%	36.60%	3.08%	8	12	20	18	14.5
DriverNet	39.38%	50.67%	59.15%	22.39%	3	9	16	3	7.75
e-Driver	36.07%	51.05%	78.85%	**28.65%**	6	8	9	**1**	6
iPAC	11.13%	29.16%	32.71%	1.38%	21	20	21	21	20.75
MSEA	13.36%	32.01%	64.81%	2.58%	20	19	14	19	18
OncoDriveCLUST	44.32%	21.61%	87.10%	19.38%	13	13	6	7	9.75
DrGap	18.81%	42.69%	88.79%	3.30%	16	14	4	16	12.5
DriverML	48.19%	70.55%	94.01%	20.38%	2	2	2	5	2.75
OncodriveFML	33.78%	48.03%	81.15%	11.02%	7	10	7	12	9
SCS	5.15%	1.32%	19.66%	0.23%	23	23	22	23	22.75
rDriver	38.18%	53.17%	87.89%	12.97%	4	5	5	10	6
UniCovEx	29.01%	55.70%	65.56%	3.52%	10	3	13	17	10.75
FI-net	**53.01%**	**88.20%**	**95.18%**	21.46%	**1**	**1**	**1**	4	**1.75**

*The bold number indicates the best result*.

The authors apologize for this error and state that this does not change the scientific conclusions of the article in any way. The original article has been updated.

