# Supplementary material for: FI-Net: Identification of Cancer Driver Genes by Using Functional Impact Prediction Neural Network
Source: Front Genet. 2020 Nov 10;11:564839. doi: 10.3389/fgene.2020.564839 (PMC7683798; doi:10.3389/fgene.2020.564839)
Supplement: Supplementary file 1 [file Data_Sheet_1.PDF]

Supplementary Material:
FI-net: identification of cancer driver genes by using functional impact prediction neural network

1 THE COMPARATIVE ANALYSIS FOR FI-NET USING MUTATIONASSESSOR AND CADD

FI-net used the local files “MA scores rel3 hg19 full” (available at `http://mutationassessor.org/r3/`) from MutationAssessor to calculate the functional impact score (FIS) for genes. In addition, other methods evaluating the functional impacts of mutations (e.g. SIFT, GERP, PolyPhen, and CADD) can also be used in FI-net. We embedded CADD to FI-net and identified driver genes in breast invasive carcinoma (BRCA), glioblastoma multiforme (GBM), acute myeloid leukemia (LAML), thyroid Carcinoma (THCA), and uveal melanoma (UVM). The CADD scores were obtained by uploading VCF files (corresponding to the MAF files) in CADD website (`https://cadd.gs.washington.edu/score`). “PHRED” of CADD was used to measure the functional impact of mutations. The overlaps between driver genes identified by FI-net using MutationAssessor and CADD are shown in Figure S1, S2, S3, S4, and S5. FI-net identified many common driver genes by using these two methods to calculate FISs for genes. Although the results of FI-net may be influenced by the prediction tools of FISs, some crucial driver genes with deleterious mutations were always predicted. For example, *EGFR, PTEN, TP53, NF1, PIK3CA* were identified in GBM; *PIK3CA, TP53, MAP3K1, GATA3, CDH1, BRCA1, BRCA2, PTEN, ATM* were identified in BRCA.

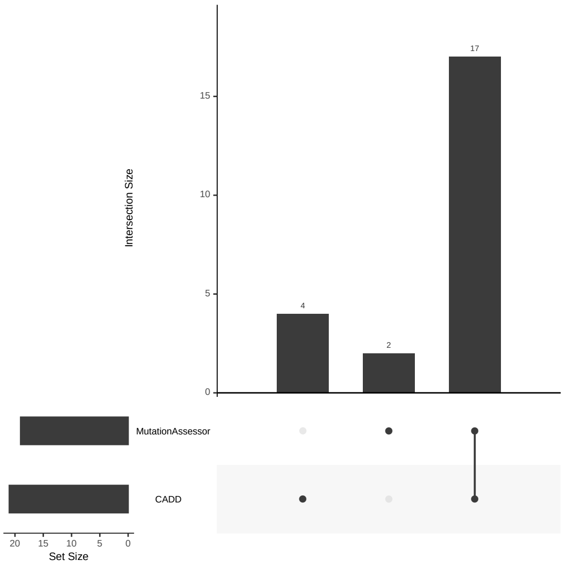

Figure S1. The overlaps between driver genes identified by FI-net in BRCA using MutationAssessor and CADD.

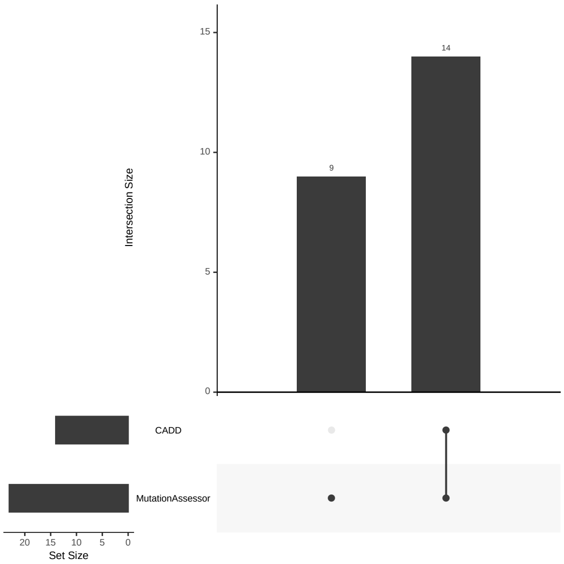

Figure S2. The overlaps between driver genes identified by FI-net in GBM using MutationAssessor and CADD.

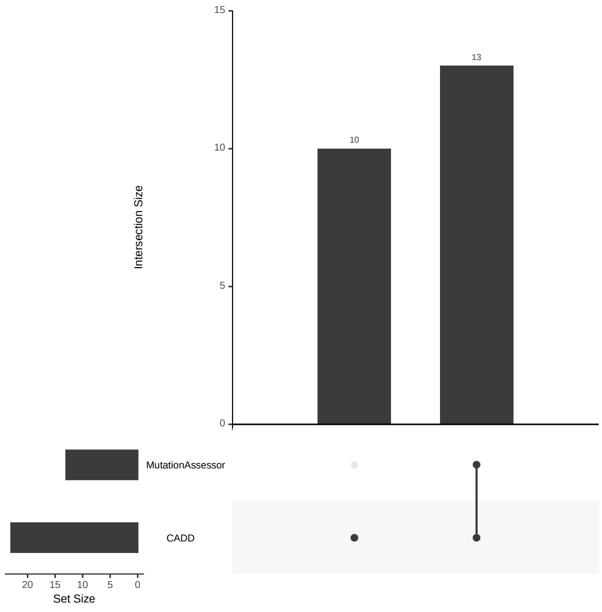

Figure S3. The overlaps between driver genes identified by FI-net in LAML using MutationAssessor and CADD.

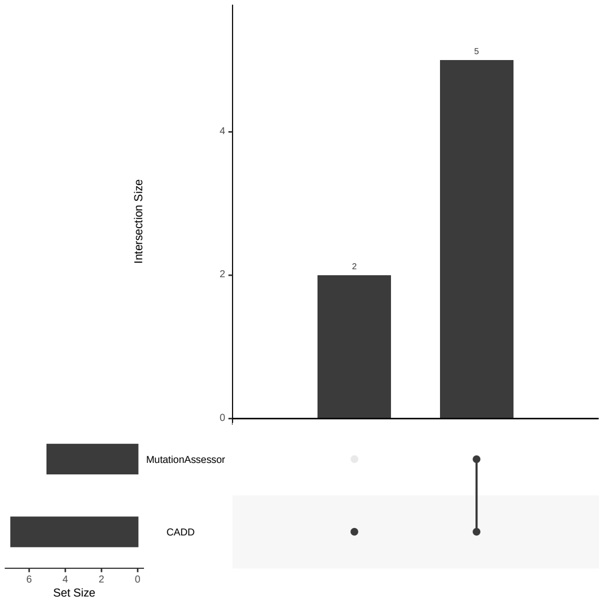

Figure S4. The overlaps between driver genes identified by FI-net in THCA using MutationAssessor and CADD.

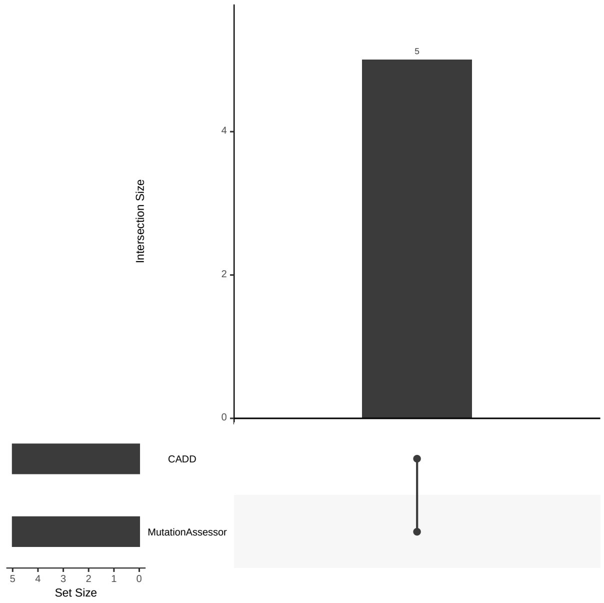

Figure S5. The overlaps between driver genes identified by FI-net in UVM using MutationAssessor and CADD.

2 THE NON-LINEAR RELATIONSHIPS BETWEEN FIS AND THE MULTI-OMICS FEATURES

Twelve genetic features from multi-omics data sources were used to estimate the FISs of genes, including the expression level, the DNA replication timing, the chromatin compartment (HiC), the length of genomic regions, the constraint score for non-synonymous mutations, the hubness in a gene expression network, the gene's known regulatory role based on gene annotation databases, the genomic copy number variation (CNA), the methylation status, the total mutation number among patients, the deleterious mutation (including mutations with null and nonsilent effects) number, and the standard deviation of functional impact score across patients. Figure S6, S7, and S8 shows the scatter plots between FIS and the multi-omics features of 300 genes (randomly sampling) in BRCA, GBM, and LAML. As shown in the scatter plots, there are non-linear relationships between FIS and the multi-omics features. In addition, we reduced the 12-dimensional multi-omics features to 2-dimensional features using t-SNE method and drew the scatter plots between the FIS and the 2-dimensional features in 3D space. As shown in the scatter plots in Figure S9, S10, S11, the FISs and the features after dimensionality reduction also have non-linear relationships.

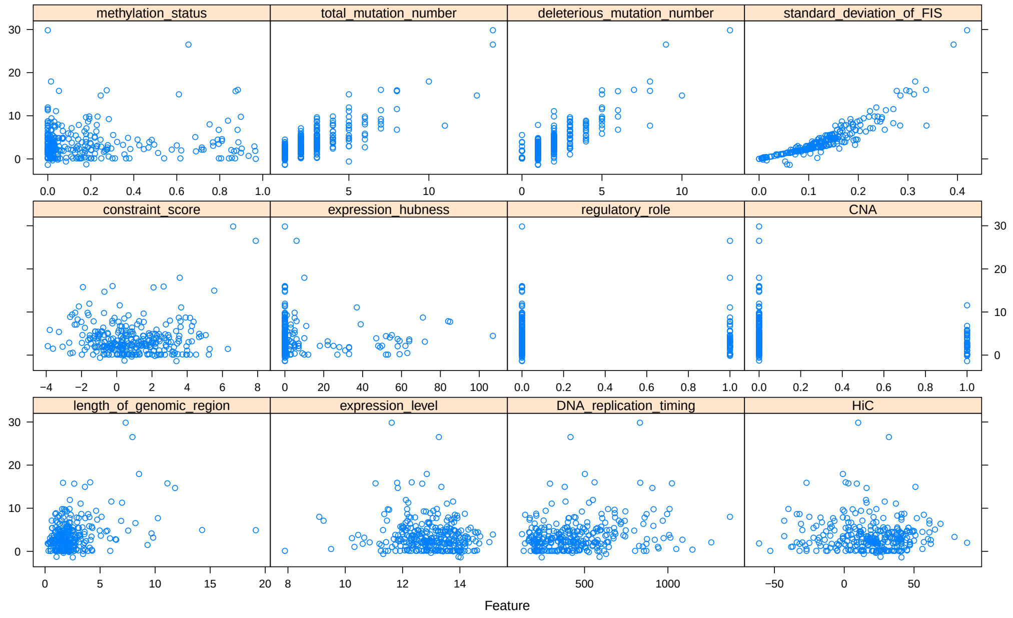

Figure S6. The scatter plots between FIS and multi-omics features of 300 genes (randomly sampling) in BRCA.

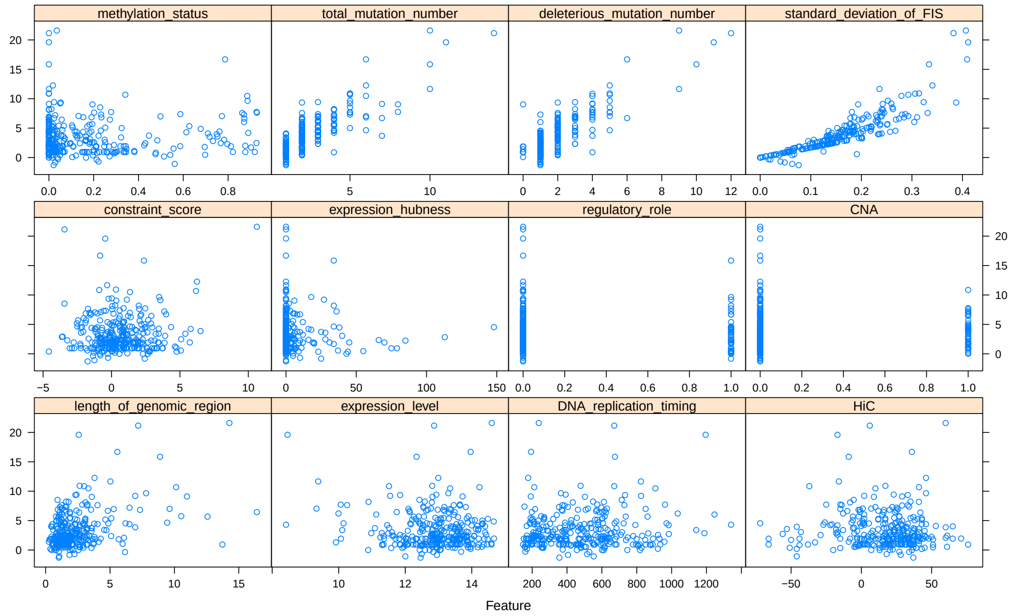

Figure S7. The scatter plots between FIS and multi-omics features of 300 genes (randomly sampling) in GBM.

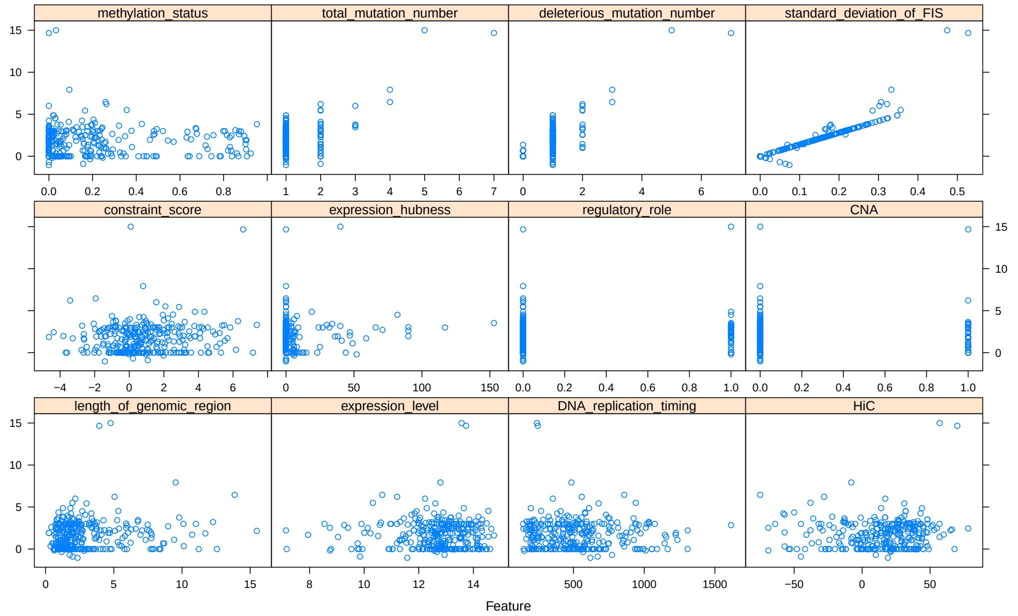

Figure S8. The scatter plots between FIS and multi-omics features of 300 genes (randomly sampling) in LAML.

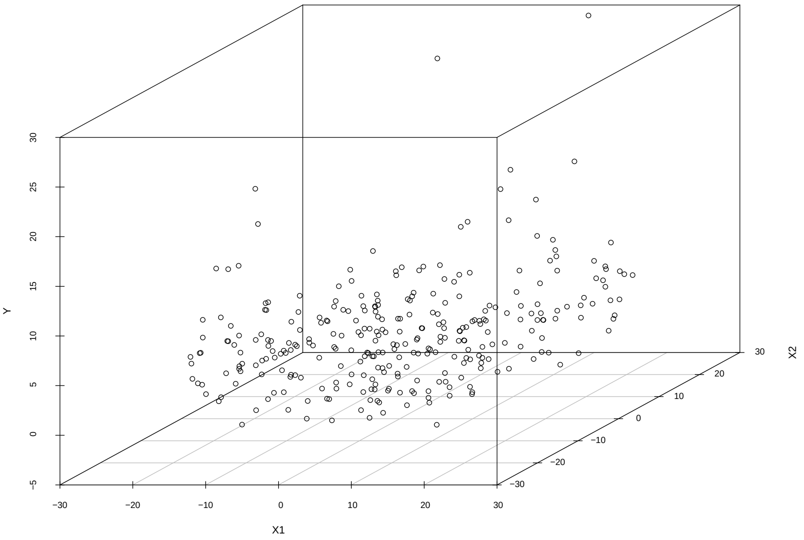

Figure S9. The scatter plots between FIS and 2-dimensional features of 300 genes (randomly sampling) in BRCA.

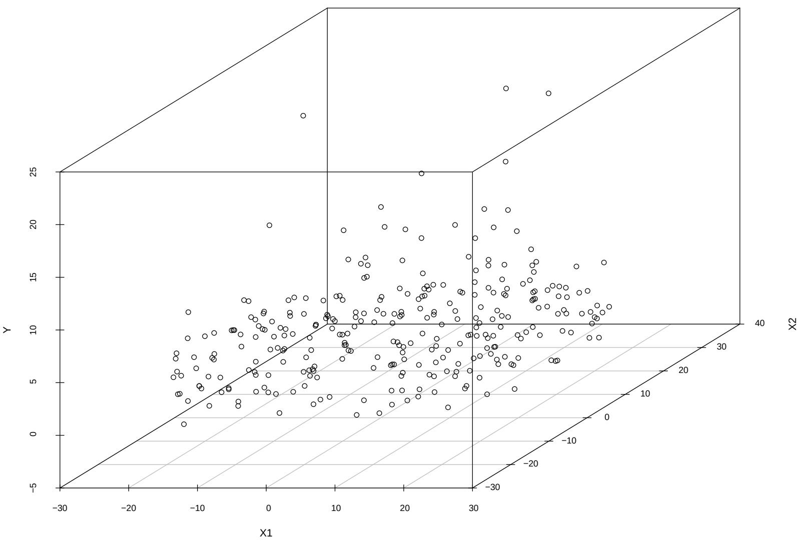

Figure S10. The scatter plots between FIS and 2-dimensional features of 300 genes (randomly sampling) in GBM.

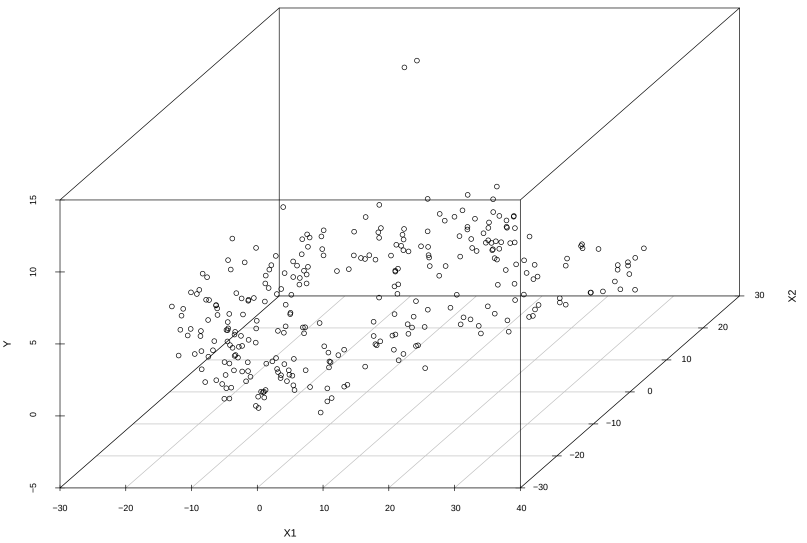

Figure S11. The scatter plots between FIS and 2-dimensional features of 300 genes (randomly sampling) in LAML.

3 THE PERFORMANCE OF FI-NET WITH DIFFERENT PRE-DEFINED EXPECTED NUMBER OF GENES

The number of clusters influences the background distribution, and hence affects the performance of identifying driver genes. The number of clusters N_c was set as the following:

$$N_c = \left\lceil \frac{G}{N} \right\rceil$$

where G is the total number of genes under study, N is a pre-defined expected number of genes in each cluster. Here, we analyzed the performance of FI-net for N being 1000, 2000, 3000, 4000, and 5000.

1. The estimated functional impact score (FIS) obey gamma distribution approximately when N is set to 1000 to 5000 in cancer datasets. Figure S12, S13, S14, S15, and S16 show the histograms of FIS in BRCA when N is set to 1000 to 5000.
2. The number of identified driver genes increases as N increases. Figure S17 shows the number of driver genes identified by FI-net when N is set to 1000 to 5000. The average number of driver genes among 31 datasets for N being 1000 to 5000 are 7, 10, 17, 20, and 23.
3. The proportion of overlap with the CGC driver list (denoted as precision) decreases as N increases. Figure S18 shows the precision of FI-net when N is set to 1000 to 5000. The average precision among 31 datasets for N being 1000 to 5000 are 55.62%, 54.39%, 53.01%, 50.81%, and 46.38%.

Based on the above analysis, we set N to 3000 to provide the appropriate number of putative driver genes for the further studies and unbiased evaluation of FI-net.

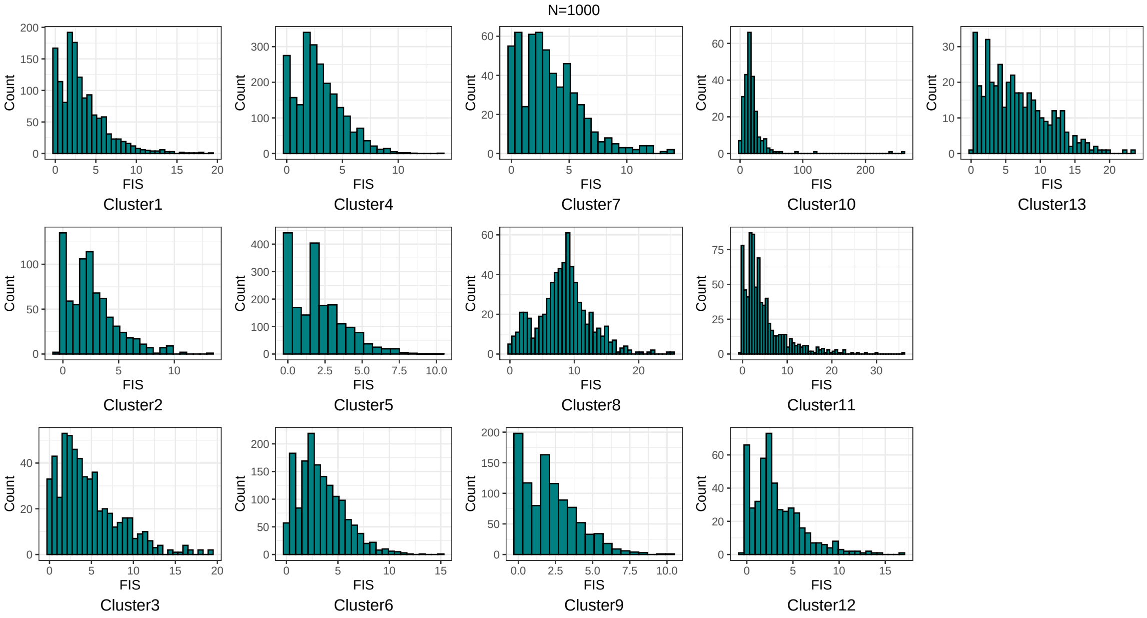

Figure S12. The histograms of FISs in BRCA for $N = 1000$.

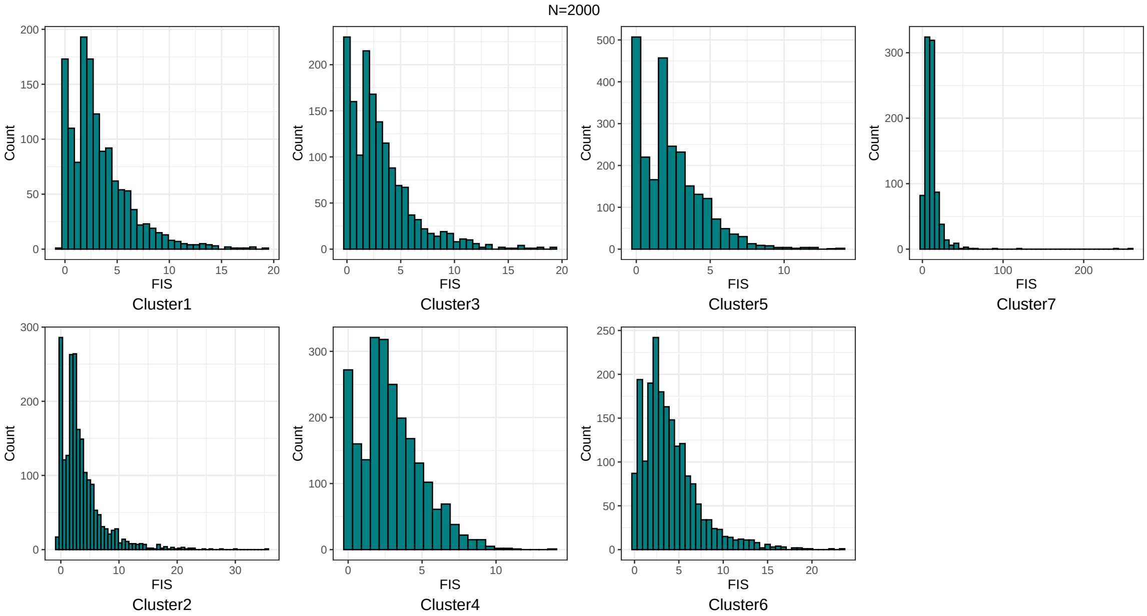

Figure S13. The histograms of FISs in BRCA for $N = 2000$.

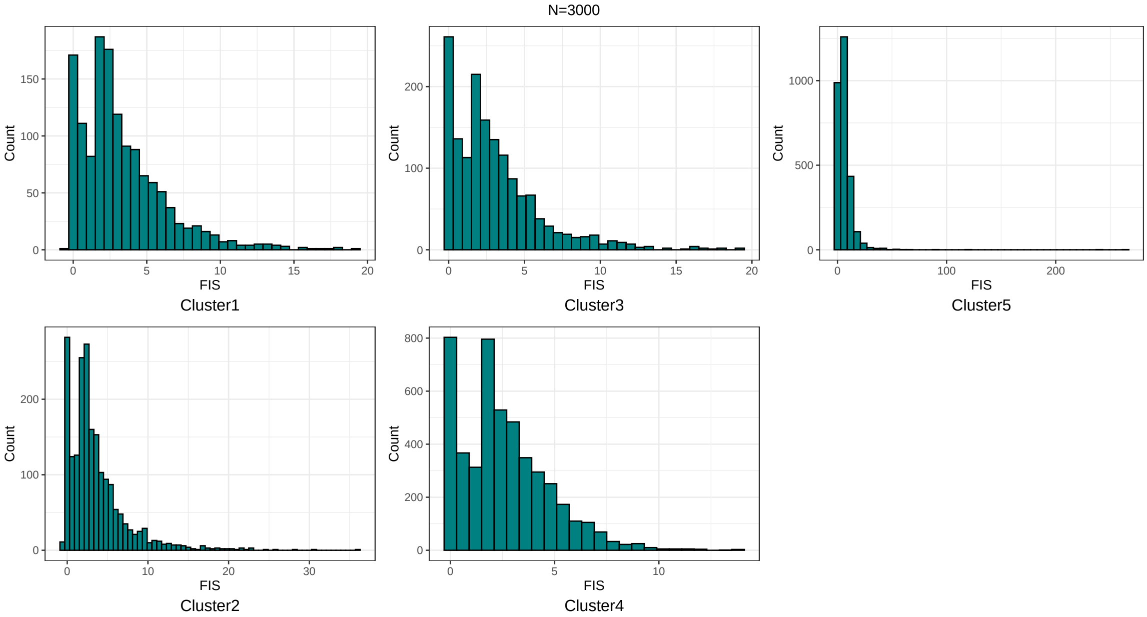

Figure S14. The histograms of FISs in BRCA for $N = 3000$.

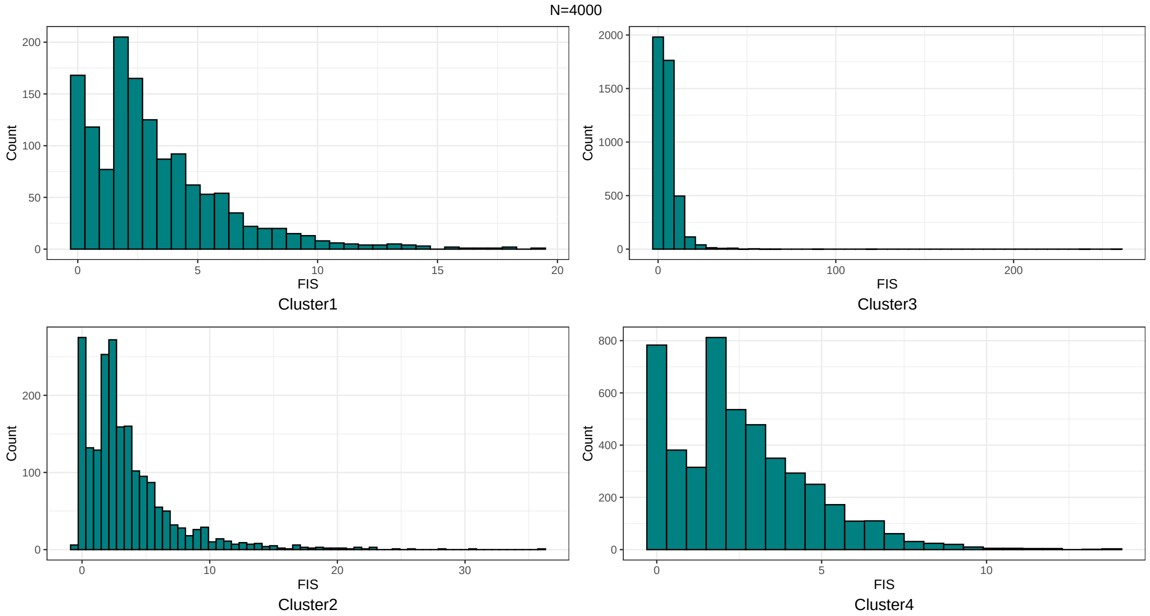

Figure S15. The histograms of FISs in BRCA for $N = 4000$.

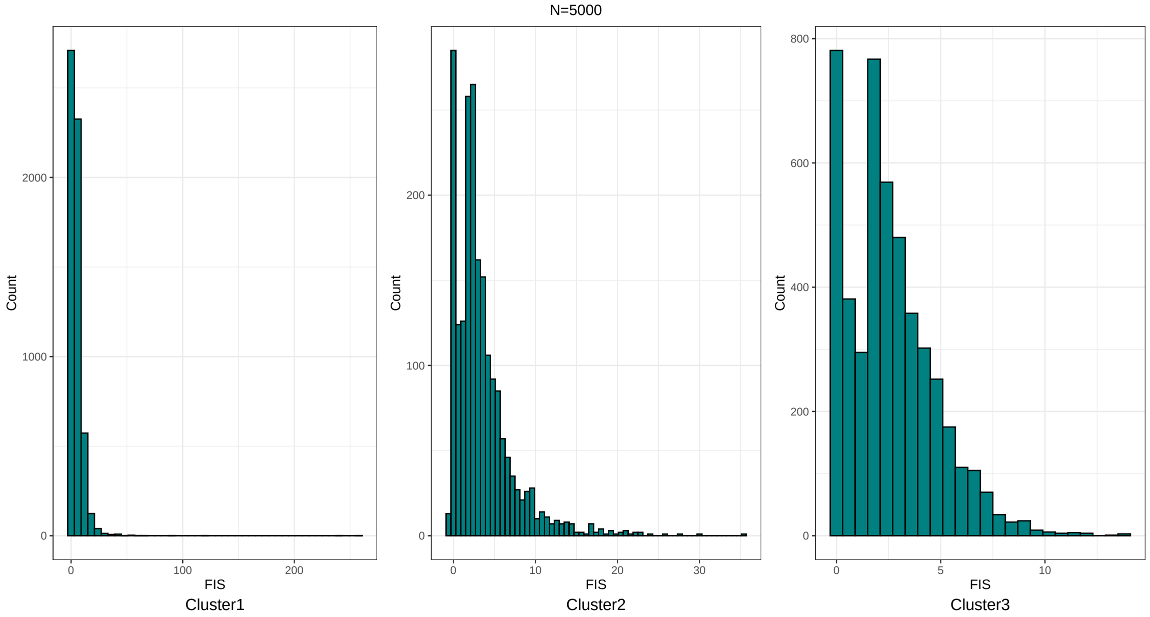

Figure S16. The histograms of FISs in BRCA for $N = 5000$.

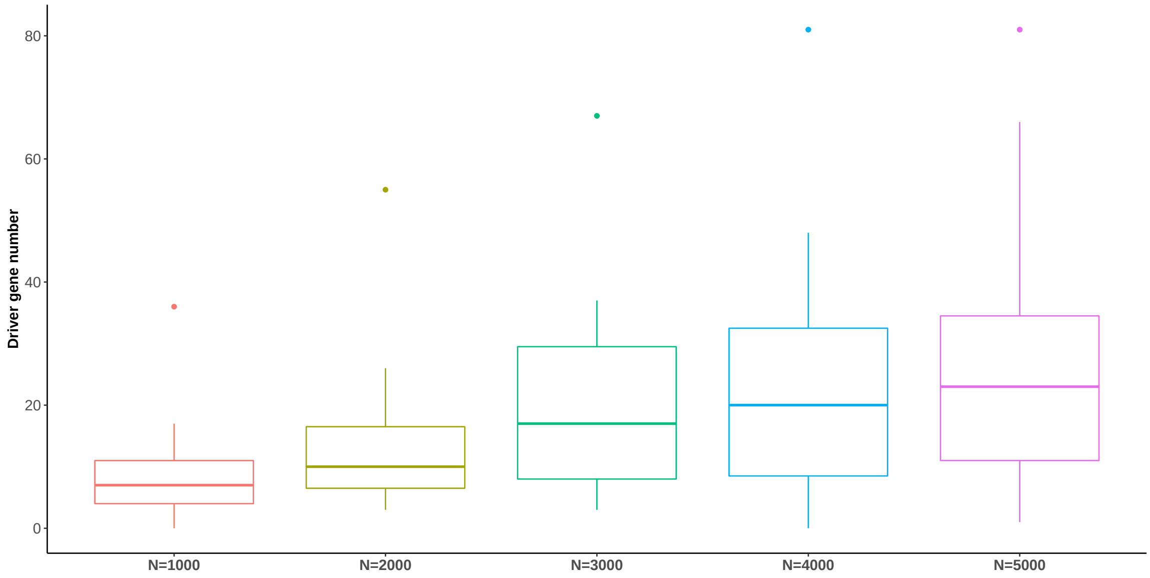

Figure S17. The number of driver genes identified by FI-net when N is set to 1000 to 5000.

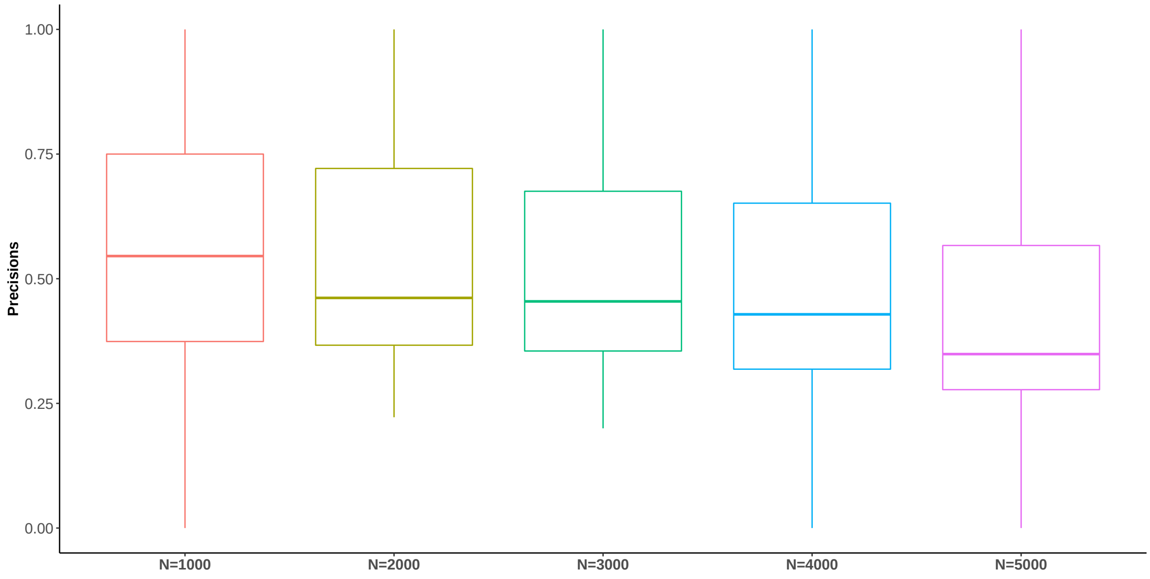

Figure S18. The precision in CGC database of FI-net when N is set to 1000 to 5000.

4 THE OVERLAP BETWEEN FI-NET AND OTHER THREE METHODS

FI-net identified some known driver genes that also predicted by other methods. The overlap between FI-net and other three newest methods OncodriveFML (functional-based method), DriverML (frequency-based method), UniCovEx (pathway-based method) in their predictions of LAML and BRCA are shown in Figure S19 and S20. As shown in Figure S19, 11 of 13 driver genes of FI-net were also predicted by at least one other method in LAML. As shown in Figure S20, 7 of 13 driver genes of FI-net were also predicted by at least one other method in BRCA.

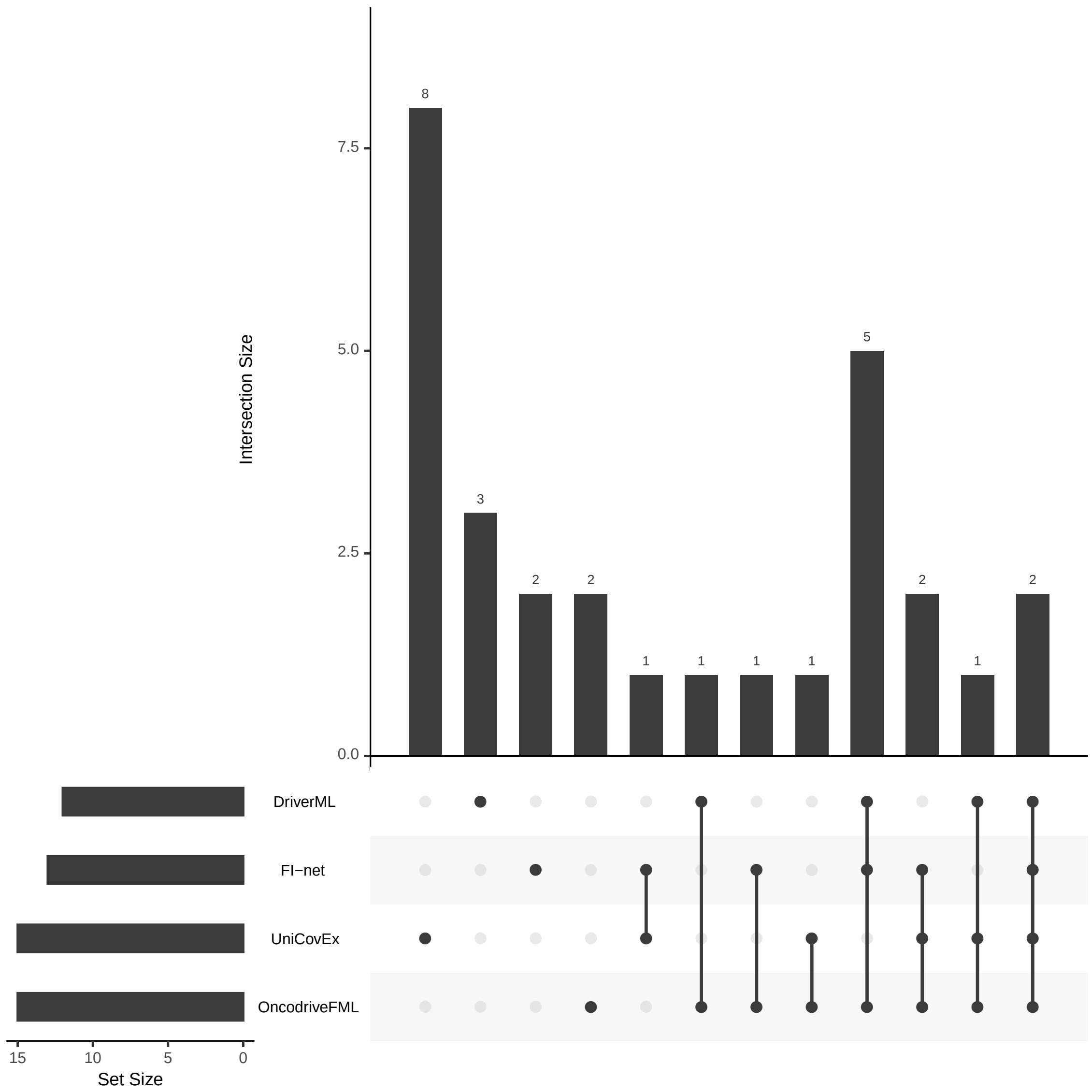

Figure S19. The overlap between FI-net and other three methods in LAML.

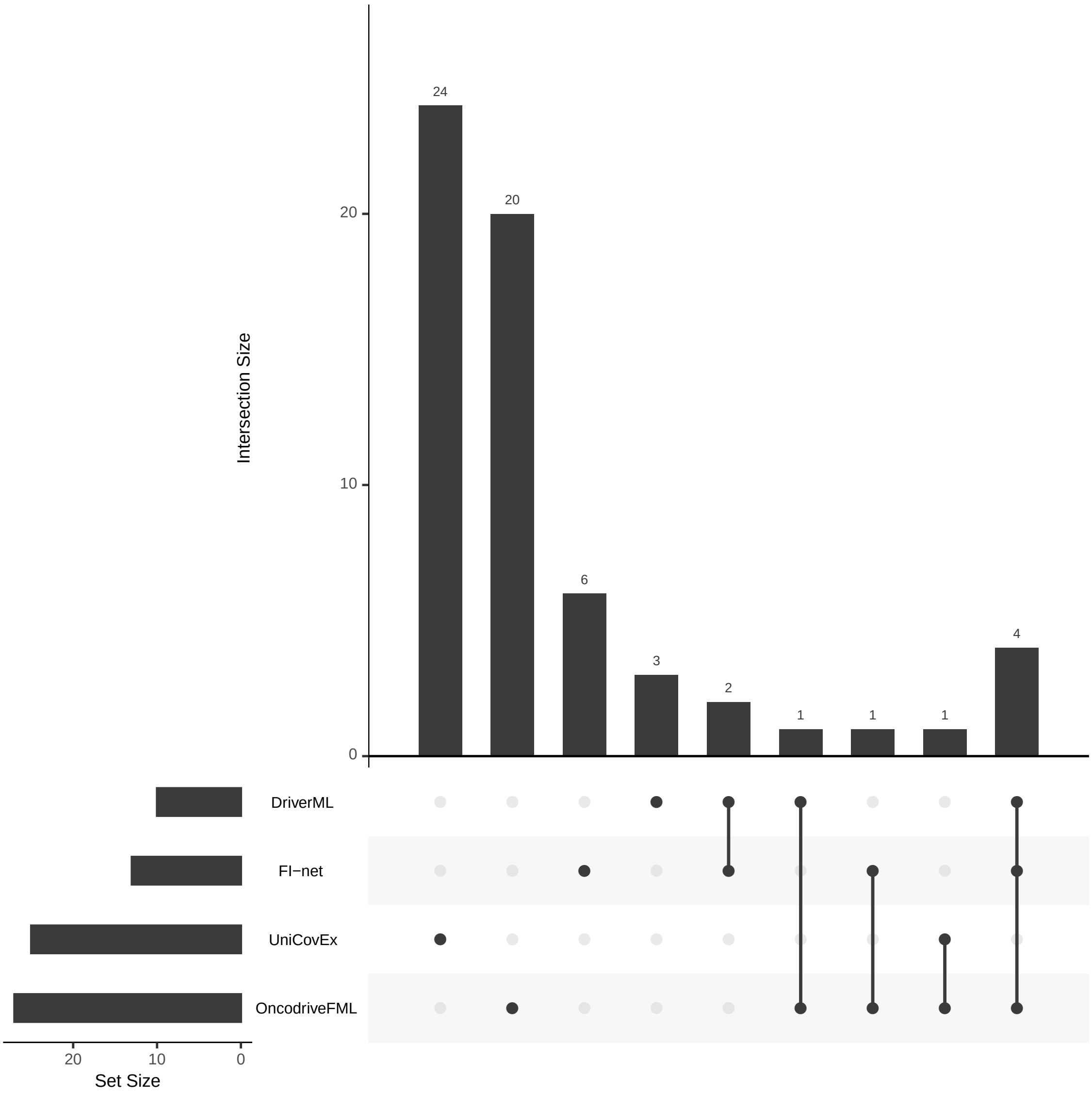

Figure S20. The overlap between FI-net and other three methods in BRCA.
